# AI literacy and competency in nursing education: preparing students and faculty members for an AI-enabled future-a systematic review and meta-analysis

**DOI:** 10.3389/fmed.2025.1681784

**Published:** 2025-11-26

**Authors:** Majeda M. El-Banna, Mirza Rizwan Sajid, Moattar Raza Rizvi, Waqas Sami, Angela M. McNelis

**Affiliations:** 1 College of Nursing, QU-Health Sector, Qatar University, Doha, Qatar; 2 Department of Statistics, University of Gujrat, Gujrat, Pakistan; 3 Faculty of Allied Health Science, Santosh Deemed to be University, Ghaziabad, India; 4 Department of Pre-Clinical Affairs, College of Nursing, Health Sector, Qatar University, Doha, Qatar; 5 School of Nursing, Vanderbilt University, Nashville, TN, United States

**Keywords:** AI literacy and competency, nursing curriculum integration, faculty readiness, ethical and institutional challenges, systematic literature review, meta-analysis, AI competence in healthcare

## Abstract

**Introduction:**

Artificial Intelligence (AI) has made its way into every dimension of human life, and its impact is significant and multifaceted. Specifically, the effect of AI in nursing education has reshaped the healthcare system. However, this technological shift in nursing and the healthcare system still needs to be evaluated in multiple aspects to ensure the effective use of AI and to prepare future professionals.

**Methods:**

This PROSPERO-registered systematic literature review and meta-analysis explored the integration of AI literacy and competency within nursing curricula and the profession globally from January 2020 to June 2025. The study specifically aimed to: (1) examine the extent of AI integration within nursing curricula; (2) assess the awareness, attitudes, and readiness of nursing students, faculty, and practitioners toward AI; (3) evaluate the effectiveness of educational interventions designed to enhance AI literacy and competency; (4) identify ethical, institutional, and pedagogical challenges associated with AI adoption in nursing education; and (5) provide evidence-based recommendations for standardized and equitable AI education frameworks in nursing.

**Results:**

The review synthesizes evidence from 111 peer-reviewed articles, including 18 distinct quantitative studies, which have been further analyzed through meta-analytic techniques. PRISMA guidelines were followed to search for relevant articles and extract the required information. Meta-analysis reveals high levels of AI-related awareness (pooled estimate = 73%, 95% CI: 64–80%) and positive attitudes (71%, 95% CI: 63–78%) among various nursing groups. The implementation of AI-related skills remains highly variable (67%, 95% CI: 55–78%), especially in low-resource settings, which needs careful interpretation. Overall, meta-analysis findings highlight significant variations and reflect non-uniformity and disparities across regions, institutions, and nursing groups (students, staff, faculty).

**Conclusion:**

Thematic synthesis underscores the need for standardized AI education, tailored faculty development, and equitable access to digital tools. Although individual-level awareness and attitudes toward AI are promising, this review reveals a lack of institutional readiness, with many nursing programs lacking standardized curricula, faculty training, and infrastructural support. Moreover, findings emphasize the critical need for broader institutional and policy efforts to match individual enthusiasm with institutional capacity in preparing nurses for an AI-enabled healthcare landscape. Further, this review offers evidence-based recommendations for various stakeholders to ensure inclusive and future-ready nursing education.

**Systematic review registration:**

https://www.crd.york.ac.uk/prospero/, identifier CRD420251090108.

## 1 Introduction

Artificial Intelligence (AI) has become an integral part of this continuously evolving era. It has transitioned from speculative potential to a defining reality of the contemporary world. It has made its impact in every facet of human life, ranging from personalized recommendations on human choices to autonomous systems (1). Its pervasiveness is redesigning not only how services are delivered but also how individuals work, perform, learn, communicate, and care for others. In the realm of healthcare, this transformation is even more evident. AI has enhanced diagnostics, administrative efficiencies, and predictive analytics that were previously unimaginable (2). As the Fourth Industrial Revolution (IR4) unfolds, equipping workers and professionals with AI literacy is becoming increasingly inevitable (3). This revolution represents the ongoing fusion of digital, biological, and physical technologies that are transforming industries and professions worldwide. It is not only relevant to technology-oriented fields but also across all human-related professions and occupations, especially healthcare (4). AI integration necessitates an urgent reevaluation of educational paradigms and frameworks in medical education, particularly for nursing staff, the cornerstone of the healthcare system. Digital knowledge and skills in the era of AI are essential in healthcare settings (5). This is to ensure that future practitioners are not merely the end-users of digital tools and technologies, but rather become informed, adaptive, and ethical participants in AI-driven systems (6).

In the field of medical and healthcare systems, integration of AI has led to transformative changes in clinical diagnostics, treatment, planning, patient outcomes, and administrative efficiency. Additionally, predictive analytics for identifying patient prognosis and deterioration, AI-enabled clinical decision support systems (CDSS), natural language processing (NLP) for patient notes, automated documentation and charting, and conversational agents used in simulation-based education are commonly employed (7). AI-powered tools can streamline workflows, reduce errors, and support evidence-based practice, empowering nurses and clinical staff to make informed decisions more efficiently and effectively. However, the implementation of these technologies might be linked with significant educational and ethical challenges, which can hinder their adoption.

While AI technologies are progressing and evolving rapidly, many educational programs remain unprepared. Research highlights that most curricula have yet to include AI-related general health informatics, such as ethics, algorithmic bias, data literacy, and critical appraisal of AI outputs (8, 9). Educators often express limited understanding, trust, and confidence in AI, which further creates barriers to incorporating AI in clinical and medical education (10). At the same time, nursing students report an increasing awareness of AI’s relevance but feel underprepared to get involved with such tools and technologies in clinical practice (11).

This gap in technology and competency to use has far-reaching repercussions. As AI becomes embedded in electronic health records (EHRs), triage systems, and monitoring devices, nurses must be able to critically analyze algorithmic recommendations, uphold ethical standards, and recognize limitations in digital interactions. Without formal training and practice, nurses may struggle to utilize AI tools or even resist their adoption altogether. Ultimately, this will result in workflow inefficiencies and potential harm to patients. Additionally, the absence of standardized AI education frameworks contributes to inconsistencies across countries and institutions. Early pilot initiatives in high-income countries such as the USA and Canada have shown positive educational outcomes, including improved student competence, confidence, and engagement with AI-assisted clinical reasoning (10–12). In contrast, low- and middle-income countries face resource constraints that impede the adoption of AI technologies in health education. The financial burden of infrastructure development, procurement of AI-enabled simulators, software licensing, and faculty training remain significant (13, 14). However, available economic evaluations indicate that, once established, AI-enhanced education can improve training efficiency and reduce long-term instructional and clinical-error costs, suggesting that the long-term benefits may outweigh the initial investment (15). Consequently, healthcare organizations are emphasizing the need for strategic, cost-effective approaches to build digital competencies and AI readiness in health education reform.

Developing AI literacy in nursing education is not merely a technical need but a sociotechnical and ethical imperative that has the potential to redefine professional practice. As AI systems increasingly impact diagnostic reasoning, workflow automation, and patient prioritization, the epistemological boundaries of nursing are being redrawn. Nurses must navigate not only digital tools but also the ethical terrains they produce, such as accountability in algorithm-informed decisions, data privacy, and equity of access to AI-supported healthcare (13, 14). Scholars have highlighted that current nursing curricula lack content related to AI technologies and their implications for clinical practice (15). A curricular transformation is needed that extends beyond tool proficiency to include ethical foresight, critical data literacy, and reflective judgment rooted in nursing’s core values of patient-centeredness and compassion (6).

Yet, as the literature suggests, few institutions have operationalized this transformation and implemented changes in their structural pedagogies. Faculty often lack institutional backing, access to the interdisciplinary expertise required for AI integration, and standardized teaching frameworks (8). Without thoughtful and deliberate evidence-based efforts to incorporate AI literacy and competency into nursing curricula, the profession risks marginalization in digital health leadership, ceding influence over the design, implementation, and governance of emerging healthcare technologies. Given the rapid pace at which AI is transforming healthcare and the lag in curricular uniform response in nursing education, a thorough systematic review of existing but recent efforts is essential to gauge the level of AI literacy and competency in the nursing profession. A comprehensive synthesis is necessary to identify and validate the potential bottlenecks that may hinder the integration of AI in nursing education. These bottlenecks often arise from structural, pedagogical, and ethical challenges that limit the seamless adoption of AI technologies within academic settings. In light of these critical gaps, the present study was guided by the following key objectives: to examine the extent to which AI-related content is embedded within current nursing curricula; to assess the perceptions and readiness levels of nursing faculty and students toward AI integration; to evaluate the effectiveness of educational interventions aimed at enhancing AI-related competencies and awareness; and to explore the challenges associated with ethical concerns and practical implementation of AI in nursing education. Specifically, it examined the global integration of AI into nursing curricula, assessed the readiness and attitudes of students and faculty, and explored pedagogical, ethical, and infrastructural challenges alongside strategies to address them.

## 2 Materials and methods

### 2.1 Study design

This study employed a mixed-methods approach combining systematic review, thematic synthesis, and meta-analysis to provide a holistic evaluation of AI literacy and competency in nursing education. This systematic literature review (SLR) and meta-analysis was registered at PROSPERO (CRD420251090108) and conducted using the Preferred Reporting Items for Systematic Reviews and Meta-Analyses (PRISMA) 2020 guidelines. The research framework SPIDER (16) (S for Sample, PI for Phenomenon of Interest, D for Design, E for Evaluation, and R for Research type) was adopted for the study to structure the review (Table 1).

**TABLE 1:** Spider framework application.

Component	Description
S	Nursing faculty, staff, and students
PI	AI literacy and competency in nursing education
D	Qualitative, mixed methods, intervention studies, reviews
E	Awareness, attitudes, readiness, skills, implementation
R	Empirical and systematic research

Each component of the SPIDER framework was clearly defined and applied during the development of the search strategy. The sample included nursing students (undergraduate and postgraduate), nursing faculty, and practicing nurses to capture perspectives across both educational and professional settings. The Phenomenon of Interest focused on AI literacy, competency, and readiness within nursing education and practice. The Design covered quantitative, qualitative, and mixed-methods studies, including both intervention and observational designs. The Evaluation element guided the inclusion of studies assessing awareness, attitudes, readiness, skills, and implementation of AI. Finally, the Research Type ensured the inclusion of empirical and systematic studies published between January 2020 and June 2025, reflecting the most recent evidence on AI integration in nursing education.

Two independent reviewers, MEB and WS, screened all titles and abstracts, followed by a full-text review to determine eligibility. Disagreements were resolved through discussion or consultation with a third reviewer (AMM). For data extraction, two reviewers independently extracted study characteristics, outcomes, and relevant variables using a standardized data extraction form. Extracted data were cross-verified, and discrepancies were resolved through consensus.

### 2.2 Inclusion and exclusion criteria and research framework

The following inclusion and exclusion criteria were applied for SLR and meta-analysis. Studies were included if they were peer-reviewed articles published between January 2020 and June 2025, focused on AI education and literacy among nurses, including nursing students and faculty, and addressed relevant outcomes or perceptions. Articles were excluded if they were opinion papers, not published in English, not fully accessible, or unrelated to nursing education.

### 2.3 Databases searched

To retrieve relevant research articles, a comprehensive search was conducted across several well-established databases, including Medline via PubMed, Web of Science, Pub-Med Central, Cochrane, Scopus, Google Scholar, Semantic Scholar, and CINAHL (via EBSCOhost). A four-level search strategy was employed, with each level progressively expanding the breadth of the search. These strategies were informed by keywords and terms identified through an extensive preliminary literature review. Level four represented the most exhaustive phase of the search process, ensuring maximum coverage of eligible studies.

Table 2 provides a step-by-step overview of the multi-level search strategy employed in this review, along with the number of articles retrieved at each stage. An initial total of 54,780 records were identified across the selected databases. The search was conducted at four progressive levels, each building on the previous one to enhance specificity and coverage. In Level 1, the search used the basic terms “Artificial Intelligence” OR “AI” AND “nursing education.” Level 2 expanded the scope by incorporating terms such as “Artificial Intelligence” OR “AI” OR “machine learning” OR “deep learning” AND “nursing students” OR “nursing faculty” OR “nurse educators” OR “student nurses” AND “nursing education” OR “nurse training.” Level 3 further refined the strategy by adding “ChatGPT” AND “clinical instructors,” along with outcome-related terms such as “competency” OR “training” OR “readiness” OR “AI literacy” OR “digital literacy.” Finally, Level 4 offered the most comprehensive search by including broader educational contexts such as “higher education,” “undergraduate nursing,” and “professional training,” combined with terms like “generative AI,” “technological proficiency,” and “informatics education,” AND attitudinal or perception-based concepts such as “attitudes” OR “perceptions” OR “technology acceptance” OR “self-efficacy.” These recent search terms were included to capture emerging literature following the introduction of large language models; however, they were applied alongside broader foundational keywords (“Artificial Intelligence,” “Machine Learning,” and “Deep Learning”) to maintain balance and avoid overrepresentation of newer studies in generative AI. For conceptual clarity, traditional AI refers to rule-based or machine learning driven applications. In contrast, generative AI encompasses models capable of creating new content such as text, images, or simulations (e.g., ChatGPT, image generators). Both forms were included to provide a comprehensive overview of AI integration in nursing education.

**TABLE 2:** Number of studies found against each search strategy.

S. No.	Search level description	Medline via PubMed	PMC	Cochrane	Scopus	Web of Science	Google Scholar	Semantic Scholar	CINAHL via EBSCOhost	Total
1	AI + nursing education (very broad)	120	1,500	20	358	300	23,060	150	150	25,658
2	+ Population (broader)	80	900	6	175	100	11,035	80	100	12,476
3	+ Competency/literacy focus	50	500	5	128	65	8,025	60	70	8,903
4	+ Full SPIDER-aligned expansion	28	351	3	119	225	6,936	42	39	7,743
	Total records identified	278	3,251	34	780	690	49,056	332	359	54,780

**FIGURE 1:**
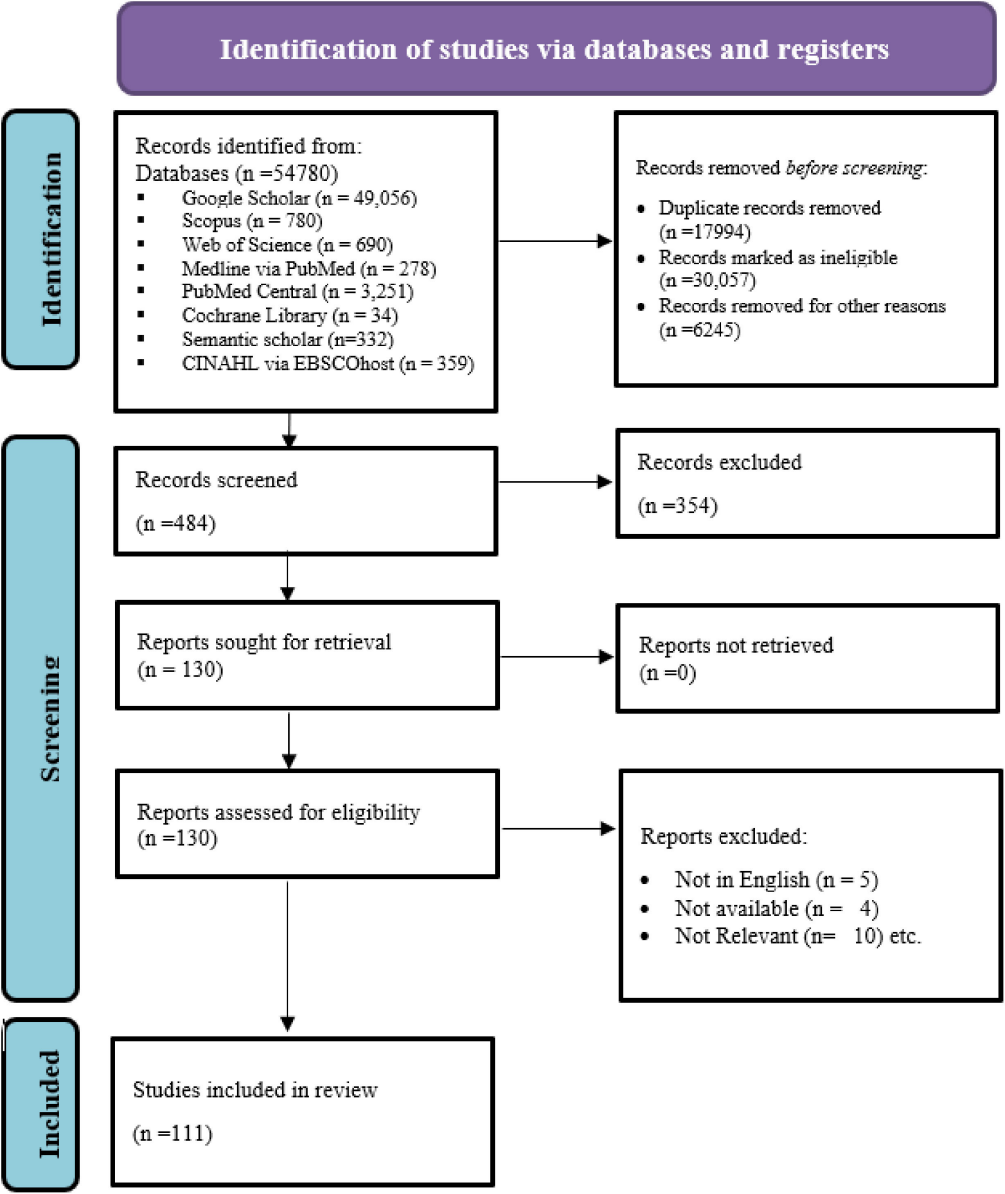
PRISMA flow diagram.

After a thorough review, 111 articles were used to conduct the SLR (Figure 1). The final articles contained information about AI, students, nursing, and curriculum, but not about other topics. Beyond any doubt, research on AI and its impact in various dimensions has been extensively noted and discussed. But in this synthesis, it’s particularly about its usage in nursing education and practice. The choice of the years is critical, i.e., 2020–2025. With the growing adoption of AI in recent years, particularly following the launch of ChatGPT, its impact on various sectors, including education and healthcare, has become increasingly evident. Barun and Clarke’s six-phase framework for thematic synthesis was followed (17). It is comprised of familiarization of data, generating initial codes, searching for themes, reviewing themes, defining and naming themes, and producing the report.

A list of included studies, along with their demographic characteristics, is presented in Supplementary Appendix A, B. The detailed search strategy for all databases is provided in Supplementary Appendix C. Supplementary Appendix B comprises the 18 studies used in the meta-analysis. After removing duplications, non-content-related records were deleted. Study quality and risk-of-bias assessments were conducted in accordance with the study design. Cross-sectional studies were evaluated using the Joanna Briggs Institute (JBI) Critical Appraisal Checklist for Analytical Cross-Sectional Studies, randomized controlled trials were assessed with the RoB 2.0 tool and mixed-method studies with the Mixed Methods Appraisal Technique (MMAT). Judgments were made independently by two reviewers across standard bias domains and summarized as Low, Moderate, High, or Unclear risk, with disagreements resolved through consensus.

### 2.4 Study quality and risk of bias assessment

**FIGURE 2:**
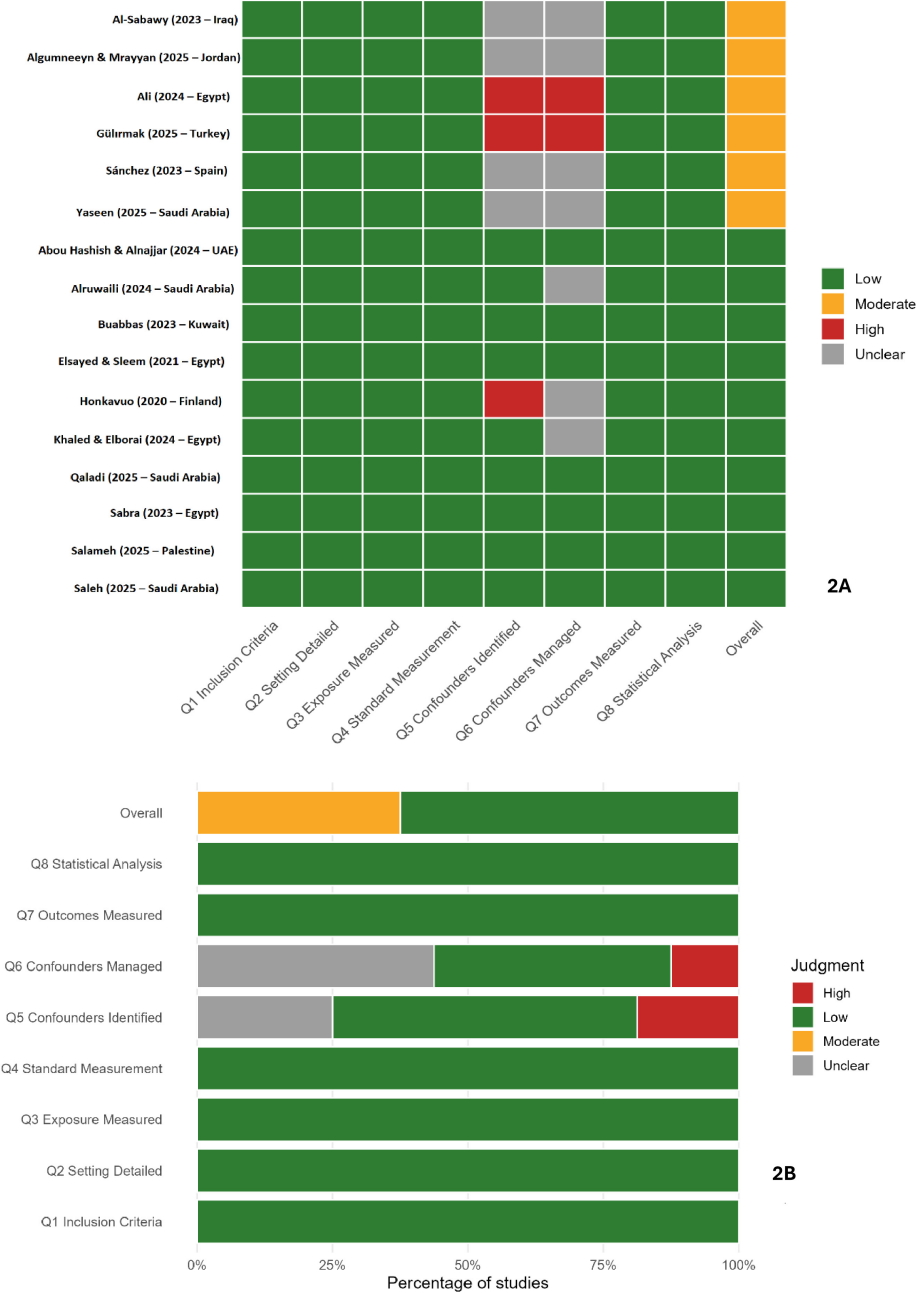
Risk-of-bias assessment for cross-sectional studies. **(A)** Traffic-light plot showing domain-wise risk-of-bias judgments for the 16 cross-sectional studies (JBI checklist). **(B)** Summary plot showing the proportion of studies rated Low, Moderate, High, or Unclear risk across JBI domains.

**FIGURE 3:**
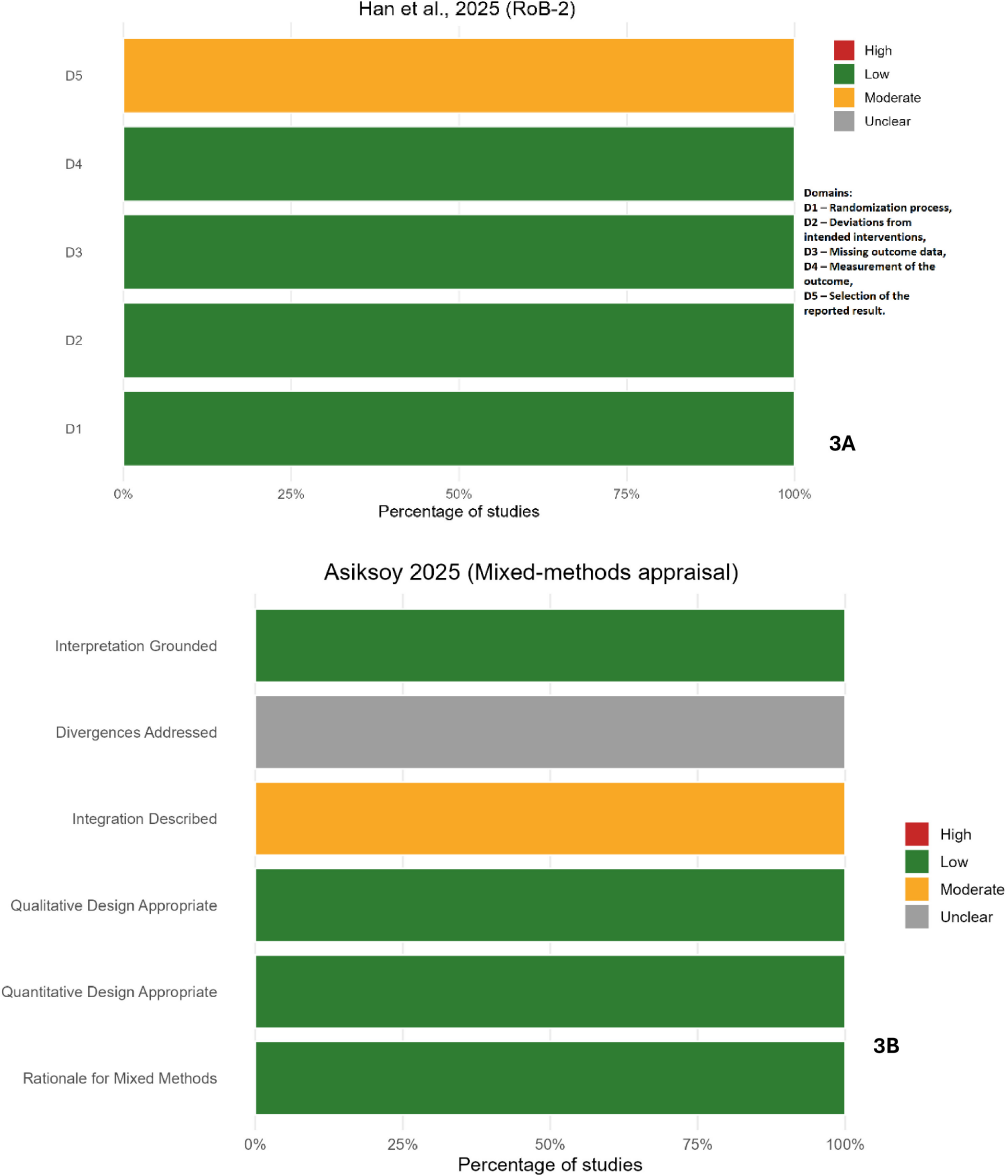
Quality appraisal for non-cross-sectional studies. **(A)** Randomized controlled trial (65) appraised with the RoB 2.0 tool. **(B)** Mixed-methods study (148) appraised with the Mixed Methods Appraisal Tool (MMAT).

Assessment of methodological quality indicated that the sixteen cross-sectional studies predominantly exhibited low to moderate risk of bias (Figures 2A, B). Most met key appraisal criteria, including clearly defined inclusion parameters, valid outcome measurements, and appropriate analytical procedures. Residual methodological limitations were primarily related to the incomplete adjustment for confounding variables and the limited representativeness of the participant samples. The randomized controlled trial demonstrated an overall low risk of bias, with minor concerns regarding allocation concealment (Figure 3A). The mixed-methods studies were both judged to be of acceptable methodological quality, showing limited integration between quantitative and qualitative components (Figure 3B). The collective appraisal suggests that the included evidence was of sound quality, lending confidence to the synthesized estimates.

### 2.5 Description of meta-analysis methodology

Eighteen (17) distinct studies were used in the meta-analysis, and the extracted data included the following headings: first author, year, title of the study, country, nursing group, sample size, and type and description of the intervention used. This extracted information was stored in Excel sheets. Later, meta-analysis was performed in R 4.5.0 (R Foundation for Statistical Computing, Vienna, Austria) using the “meta” and “metafor” libraries. Studies reported three aspects of AI-related awareness, attitude, and implementation in terms of percentages, which are equivalent to prevalence in this study. The extracted prevalence was pooled using common and random-effects models, considering the heterogeneity of individual study findings. The 95% confidence intervals (CI) were also computed for pooled findings. Heterogeneity was assessed through Q, I^2^, Tau^2^, and *p*-value. Those findings, which showed a strong and significant presence of heterogeneity (*p* < 0.05), were pooled through the random effect model. Pooled estimates and heterogeneity analysis findings were presented in a Forest plot, which is renowned for its comprehensiveness. Publication bias was evaluated through the Funnel plot and Egger’s test. Funnel plot inspection often suggests asymmetry and can lead to subjective and biased findings. Therefore, to augment the graphical view, Egger’s test was used, along with its p-value. Sensitivity analyses were also conducted to assess the robustness of findings. Meta-regression analysis was also executed to investigate the effects of sample size, nursing groups, year of publication, and country of study on pooled estimates. Further, nursing groups were considered an important factor to perform subgroup analysis, as that variable might change the mean percentages of AI-related awareness, attitudes, and implementation. The details of the included studies, along with their basic characteristics, are presented in Supplementary Appendix B.

## 3 Results

### 3.1 Thematic synthesis

The first three research questions were answered using thematic synthesis. As several included studies addressed both traditional and generative AI tools, the findings were synthesized collectively rather than stratified by AI type, allowing for an integrated interpretation of the educational implications. Digital knowledge and skills in the era of AI are essential in healthcare settings (5). To analyze the existing literature, we employed thematic synthesis, a qualitative approach that systematically identifies, analyzes, and reports patterns across studies. We have inductively identified and synthesized key themes from peer-reviewed studies published between January 2020 – June 2025 using Braun and Clarke’s six-phase framework (16). Through this process, five dominant and interconnected themes emerged: (1) curriculum integration; (2) faculty readiness; (3) student perception; (4) innovative pedagogical approaches; and (5) ethical considerations. These interconnected themes highlight systematic challenges and opportunities essential to preparing nursing students, staff, and faculty for an AI-enabled healthcare environment. Most studies viewed the use of AI positively, although some also raised concerns among nursing students and faculty members.

#### 3.1.1 Theme 1: curriculum integration of AI in nursing education

This theme establishes the foundational role of curriculum design and serves as a lens for understanding systematic gaps in AI literacy within nursing education. A significant number of articles highlighted the pressing need to incorporate AI content into nursing curricula in a structured and standardized manner, emphasizing that the absence of cohesive curricular planning restricts the development of essential digital competencies. The inclusion of AI content nurtures vital digital skills, enabling students to analyze clinical data, engage with machine learning applications, and utilize AI-enhanced decision support systems (15), thereby bridging the gap between technological knowledge and bedside decision-making. However, AI education is often fragmented, typically limited to elective modules or broader informatics courses that do not engage deeply with AI principles or practice, and many programs continue to function without clear curriculum guidelines (8, 18, 19). To achieve effective integration, curricula must be aligned with evolving healthcare technology needs and ensure coherence between theoretical content and practical competencies, supported by a shift from traditional teaching approaches to digital platforms, simulations, and AI-based learning environments (20–22). Equally critical is the influence of nursing students’ and faculty members’ perceptions of trust in AI, their digital literacy, and ethical awareness, which affect how such curricular reforms are implemented (11, 23, 24). Embedding AI into the curriculum enhances nursing students’ adaptability and efficacy in using AI for patient care, while also fostering accountability and compassionate decision-making in technology-supported clinical contexts (11).

Studies consistently emphasize that AI should be integrated into nursing education programs as a core, rather than an optional, component to prepare graduates for leadership in digitized healthcare environments (18, 21). Yet, regional disparities persist due to a lack of policy-level direction and institutional incentives for curriculum reforms, with technologically advanced nations advancing faster than resource-limited ones (15, 18, 19, 25). De Gagne offered a global perspective, emphasizing that countries leading in AI research, such as China, South Korea, and several in the European Union, were actively integrating AI training within national educational frameworks to cultivate future-ready healthcare professionals (25). In contrast, many nursing programs in countries such as Palestine, Pakistan, and the United Arab Emirates lag due to a lack of policy-level direction and institutional incentives for curriculum reform. It was recommended that nursing education should integrate AI content to educate students more effectively. Scholars have highlighted that the current nursing curriculum lacks content related to AI technologies and their implications for clinical practice (15). To address this inequity, innovative approaches in nursing education are critical for equipping students with digital competence, including the design of AI-focused modules, workshops, and experiential learning sessions that build confidence and literacy. Training courses in AI will enhance the digital literacy and competence of nursing students, and if this crucial gap persists, future nurses will be underprepared for AI-enabled healthcare systems (26, 27).

#### 3.1.2 Theme 2: faculty readiness and institutional barriers

This theme focuses on educators’ competence, confidence, and institutional support required for effective AI teaching, which is central to the successful integration of AI into nursing education. Faculty readiness was a significant concern, as AI in nursing education faces common challenges, including inadequate infrastructure, high costs, and a shortage of trained professionals in the field (28–30). A lack of foundational knowledge of AI tools among faculty members limits their ability to embed AI within both classroom and clinical training. Educators often lack the confidence to effectively engage with AI technologies because of their rapidly changing and evolving nature (31), reinforcing the need for structured professional development. Studies highlight that students require structured opportunities to use AI through simulation and real-world case examples (11, 18), which demands that faculty be equipped with the technical and pedagogical expertise needed to supervise such learning interventions (25, 32) effectively. The absence of faculty development programs specific to AI was also a recurring concern, as many instructors lack sufficient AI literacy to implement AI-based tools (33) successfully. Therefore, nursing education programs need tailored, ongoing, and continuous training that addresses both technical competencies and the ethical dimensions of AI integration (11). Institutional support was inconsistent, often due to limited leadership engagement and lack of clear policy direction, which reduced motivation to adopt or revise course structures (30, 34). Without targeted investments in infrastructure, mentorship, and policy frameworks, faculty readiness for AI integration remained critically underdeveloped (35, 36).

Furthermore, faculty attitudes and adoption behaviors are shaped by their perceptions of technology and generational differences (37–41). Younger faculty, often digital natives, exhibit greater comfort and curiosity, while older educators may be more cautious, influenced by limited, poor exposure to AI tools. The ageing of the faculty workforce further slows adoption and underscores the urgency for mentoring and cross-generational collaboration (37, 38). Some faculty members view AI as beneficial for streamlining tasks such as grading and feedback (39), whereas others fear that it undermines academic integrity or traditional pedagogical authority (40). Institutional messaging plays a decisive role when AI is framed as a collaborative tool rather than a replacement; receptivity increases (41). Conversely, top-down technology implementations, without faculty engagement, often lead to disengagement or performative compliance (42), highlighting the importance of transparent dialogue and co-creation of AI adoption strategies with faculty. Persistent infrastructural problems such as insufficient AI-compatible devices, unstable internet, and lack of advanced learning platforms impede practical integration, and with the digital divide (25, 30, 34). Many institutions still treat AI as optional content, leaving students underprepared for AI-enabled clinical environments. To overcome these limitations, faculty learning communities, innovation hubs, and mentorship initiatives have been proposed as sustainable models for capacity building (43). Finally, AI should be utilized in nursing education in a manner that is inclusive and equitable (25). When responsibly applied, AI can make learning more personalized, engaging, and effective, but this transition is both a technological and moral transition requiring reflection on values, ethics, and pedagogy. Overall, this theme highlights that faculty readiness depends on the synergy of professional development, leadership engagement, and institutional investment.

#### 3.1.3 Theme 3: student readiness and AI competency

Student readiness and AI competency, like faculty, are also vital in nursing education. Students, although digitally native, often feel underprepared to engage with AI in clinical contexts. El Arab et al. depicted that students are aware of AI’s growing influence but express limited self-efficacy and concerns about ethical responsibility (11). Findings suggest a need to understand the limitations of AI fully. Shen et al. found that nursing students in China, for instance, were more receptive to AI when they were taught through experiential learning models rather than abstract theory (18). These findings, supported by other studies (44), show that students’ positive attitudes toward AI and experiential learning enhance their acceptance and future utilization of AI technologies. Students demonstrated that moderate AI readiness implies a foundational level of cognitive understanding and ability, indicating strong potential for further development and improvement. Abou Hashish and Alnajjar highlighted that improving digital literacy can increase students’ AI competence, enabling them to respond appropriately in clinical practices (26). Nursing students generally perceive digital transformation and AI as user-friendly and beneficial in healthcare settings (26, 45), and greater awareness of AI is associated with increased competency (46).

#### 3.1.4 Theme 4: innovative pedagogical approaches and AI tools

Student perspectives serve as a valuable foundation for designing innovative pedagogical models that enable meaningful and practical AI-based learning. The incorporation of AI into nursing education represents a significant shift toward more creative and learner-centered pedagogical approaches. Innovative pedagogies enhance engagement and foster skills for clinical decision-making in AI-supported environments (32). This theme highlights instructional methods and AI tools that translate conceptual understanding into practice, where advanced simulations and adaptive learning platforms, and AI-driven analytical tools help students manage complex clinical scenarios, forecast health needs, and optimize resource planning. Innovative strategies, such as simulation-based learning, flipped classrooms, and conversational AI, enhance nursing education (32) and encourage critical engagement with emerging digital tools. AI-driven simulation platforms create realistic and immersive learning environments, allowing students to practice clinical reasoning in safe settings. Chatbots support simulation-based training and assessment (37), while adaptive systems and virtual teaching assistance deliver customized learning experiences aligned with students’ needs (47). Many scholars have highlighted the improvement in students’ skills when using simulation in their clinical practices (48, 49). Simulation and virtual teaching interventions have consistently improved students’ clinical skills and learning outcomes (48–51). However, their effectiveness depends on faculty readiness, infrastructure, access, and ethical integration. Overall, a thoughtful, evidence-based implementation of AI in nursing curricula enhances engagement, promotes competence, and prepares future nurses to excel in technologically advanced healthcare environments.

#### 3.1.5 Theme 5: social and ethical implications of AI

Ultimately, all technological and pedagogical advancements in education must be situated within a framework of ethical, social, and legal responsibility to ensure the just and equitable integration of AI (52). The incorporation of AI in nursing brings substantial ethical concerns, particularly regarding data privacy, accountability, and algorithmic bias. Recent literature consistently reports that data privacy and accountability in AI systems are not always addressed (51), underscoring the need for strong ethical foundations within AI-integrated curricula (25). Scholars caution against algorithmic bias, data misuse, and over-reliance on AI at the expense of human judgment (13, 53), emphasizing that ethical training is essential to AI readiness (54). AI systems collect sensitive data, such as academic records, and without strong security, this data is vulnerable to misuse (54). Students must understand how their data is collected, stored, and used (47), and institutions must ensure regular bias checks to maintain data integrity (55). Importantly, AI should be viewed as a supportive tool, not a replacement for mentorship, empathy, or ethical reasoning (56). Moreover, faculty readiness continues to act as a gatekeeper for curricular change (14), reinforcing the centrality of educator capacity in ethical AI adoption. The digital divide remains a critical equity issue, as limited access to infrastructure in low-resource settings restricts exposure to AI tools. Institutions must ensure inclusive access and strong data protection frameworks (54) to promote transparency and trust. Ethical standards must be applied in a humanistic, not mechanistic, manner (13), balancing technical competency with moral and critical reasoning (33, 47).

Collectively, five themes present a cohesive narrative demonstrating that AI integration in nursing education requires coordinated efforts across curriculum reform, faculty capacity building, student empowerment, pedagogical innovation, and ethical governance. A concise synthesis of the thematic pillars, core goals, strategies, and stakeholders is provided in Table 3, and an operational policy-and-governance blueprint to enact these pillars is outlined in Table 4. This multidimensional approach will ensure that the future nursing workforce is equipped with digital proficiency, ethical sensitivity, and clinical acumen for an AI-driven healthcare environment.

**TABLE 3:** Thematic pillars of AI-integrated nursing education and concerned stakeholders.

Sr. no.	Thematic pillar	Core goal	Key strategies	Primary stakeholders
1.	Curriculum integration of AI in nursing education	Embed AI into nursing curricula with interdisciplinary and clinical relevance	• Develop core/elective AI modules • Integrate informatics & ethics • Align with clinical practice needs	• Faculty • Institutions • Regulatory Authorities
2.	Faculty readiness and institutional barriers	Equip educators and institutions with skills and support for AI adoption	• Provide CPD & AI training • Foster interdisciplinary teaching • Invest in infrastructure & resources	• Faculty • Institutions • Regulators
3.	Student readiness and AI competency	Build student capacity in AI tools and ethical decision-making	• Use simulations & real-world cases • Teach AI ethics & data bias • Assess AI literacy as a learning outcome	Students Faculty Institutions
4.	Innovative pedagogical approaches and AI tools	Use AI-enhanced teaching tools to improve learning outcomes	• Integrate chatbots, virtual tutors • Apply learning analytics • Evaluate impact regularly	• Faculty • Institutions • Tech Developers
5.	Ethical and social implications of AI	Embed social responsibility and ethical reflection in AI education	• Teach ethics across the curriculum • Address data privacy & justice • Engage the community and public trust	• Students • Faculty • Society • Regulatory bodies

**TABLE 4:** Integrative layer: policy and governance mechanisms.

Sr. no	Support mechanism	Purpose	Relevant entities
1.	Policy frameworks	Ensure ethical, standardized AI integration in curricula	Ministries, nursing councils, AI experts
2.	Accreditation and standards	Institutionalize AI literacy benchmarks	Accreditation bodies, nursing boards
3.	Global collaborations	Enable knowledge exchange and alignment with international best practices	WHO, ICN, academic networks

These results present a range of consequences for all the stakeholders, including nursing instructors, the academic system, policy makers, and health system administrators:

- AI integration in the education plan: There is ample justification for teaching AI concepts at all levels of nursing education, from undergraduate to graduate level, including simulation, training chatbots, and AI-assisted decision-making frameworks.
- Change in educator role: Nurse educators require support through curricula, materials, and workshops to become competent in AI topics and should also undergo tutor training.
- Nurse roles redefined: Training must be ongoing, self-paced, easily accessed, and formally recognized. In addition to AI technologies, it should critique on workload, care, and professional identity.
- Making assured choices: The COVID-19 pandemic underscored the urgent need for evidence-based regulatory frameworks, particularly where policies for AI and digital tools were lacking. Clear guidance is needed to help practitioners balance technology and personal judgment when working with robots or AI in healthcare.
- Standardization and guidelines: Because of the variability found in this review, coherent policies on AI incorporation into nursing education and practice should be developed, incorporating local frameworks while aligning with global standards.

### 3.2 Meta-analytical findings on AI literacy and competency in nursing education

As previously mentioned, 18 distinct studies were utilized in the quantitative Meta-analysis. Within the 18 studies, 16 measured aspects of AI-related awareness, 16 measured attitude, and 13 measured implementations. The analysis provides the pooled estimates of nurses, nursing students, and nursing faculty AI-related awareness, attitudes, and implementation separately.

#### 3.2.1 Findings of meta-analysis for AI-related awareness in nursing-related groups

**FIGURE 4:**
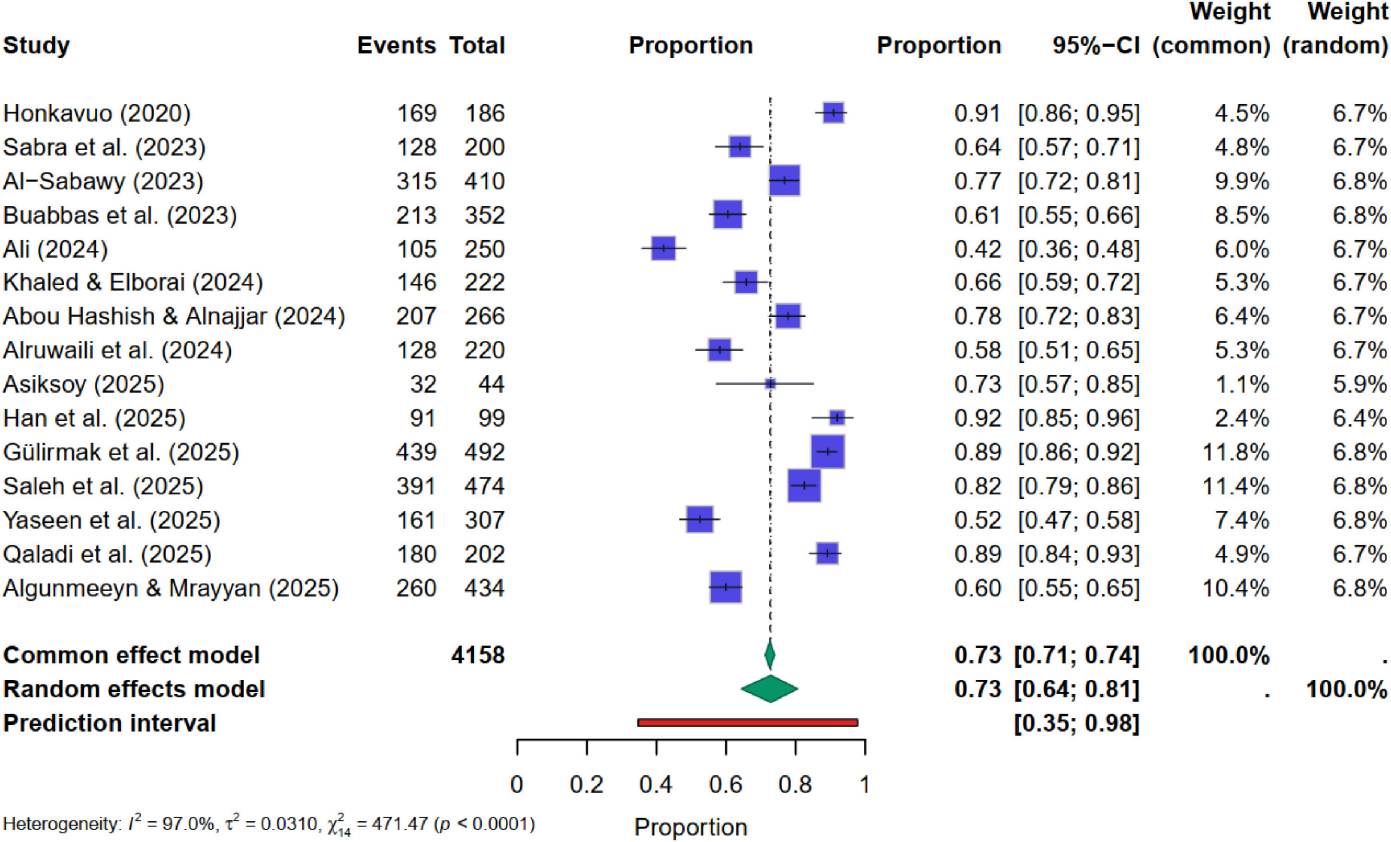
Forest plot for pooled estimates of AI-related awareness in nursing.

Figure 4 presents the forest plot, indicating a high level of heterogeneity. Q-statistic = 471.47 (*p* < 0.0001), I^2^ was 97%, Tau^2^ = 0.0310 with *p*< 0.05. Therefore, a random effect model was used to pool the study findings. Pooled estimates computed through the random-effects model showed that the average AI-related awareness ranges from 64 to 81%, with a point estimate of 73%. Results suggest that nursing students and faculty demonstrated a strong overall level of awareness related to AI. However, the wide confidence interval and high heterogeneity indicated that this awareness is not uniformly distributed in the study groups. Egger’s test yielded an insignificant regression intercept of β_0_ = −0.0516 with *p* = 0.960. This *p*-value confirms the absence of strong publication bias in selected studies (Figure 4).

Findings from the meta-regression showed that the year of publication, sample sizes of the included studies, and nursing-related groups did not significantly impact the pooled estimates of AI-related awareness. All regression coefficients with their *p*-values are reported in Table 5. *P*-values indicated the insignificance of these characteristics for AI-related awareness. However, subgroup analyses were also performed, and separate summary pooled random estimates were computed considering the nursing-related groups and their heterogeneity. These findings are presented in Table 6. Results show nursing students (79%) and faculty (82%) have significantly higher levels of AI-related awareness than nursing staff working in various hospitals (65%). The Q-statistic and its *p*-value revealed a statistically significant difference among these three proportions.

**TABLE 5:** Meta regression findings for AI-related awareness, attitude, and implementation.

Variables	Estimate	SE	Z-statistic	*p*-value	Lower limit	Upper limit
**AI-related awareness**						
Year	−0.0248	0.1760	−0.1407	0.8881	−0.3697	0.3202
Sample size	−0.0020	0.0018	−1.0827	0.2790	−0.0055	0.0016
Nurses	−0.7785	0.4661	−1.6701	0.0949	−1.6921	0.1351
Nursing faculty	0.5654	1.0046	0.5628	0.5736	−1.4037	2.5344
**AI−related attitude**						
Year	0.2437	0.1236	−1.9719	0.0486*	−0.4859	−0.0015
Sample size	0.0025	0.0015	1.7366	0.0825	−0.0003	0.0054
Nurses	−0.5122	0.3598	−1.4235	0.1546	−1.2174	0.1930
**AI-related implementation**						
Year	0.0245	0.2174	0.1127	0.9102	−0.4015	0.4505
Sample size	−0.0017	0.0023	−0.7327	0.4638	−0.0063	0.0029
Nurses	−1.0694	0.6265	−1.7070	0.0878	−2.2972	0.1585

*Statistically significant at *p* < 0.05.

**TABLE 6:** Subgroup analyses for AI-related awareness, attitude and implementation.

Group	No. of studies	Point estimate	Lower limit	Upper limit	I^2^	Tau^2^	Q-test	*p*
**AI-related awareness**								
Nursing students	8	0.7912	0.6847	0.8810	96.8%	0.0279	7.57*	0.027
Nurses	7	0.6578	0.5280	0.7767	96.6%	0.0300		
Nursing faculty	1	0.8249	0.7893	0.7893	**–**	**–**		
**AI-related attitude**								
Nursing students	10	0.7465	0.6524	0.8304	96.1%	0.0259	2.38	0.123
Nurses	6	0.6330	0.5162	0.7425	95.9%	0.0198		
**AI-related implementation**								
Nursing students	8	0.7800	0.6282	0.9013	98.0%	0.0547	3.87*	0.049
Nurses	5	0.5596	0.3944	0.7185	97.8%	0.0336		

*Statistically significant at *p* < 0.05. I^2^, percentage of total variation across studies due to heterogeneity; Tau^2^, between-study variance; Q-test, Cochran’s Q statistic for heterogeneity.

#### 3.2.2 Findings of meta-analysis for AI-related Attitude in nursing-related groups

**FIGURE 5:**
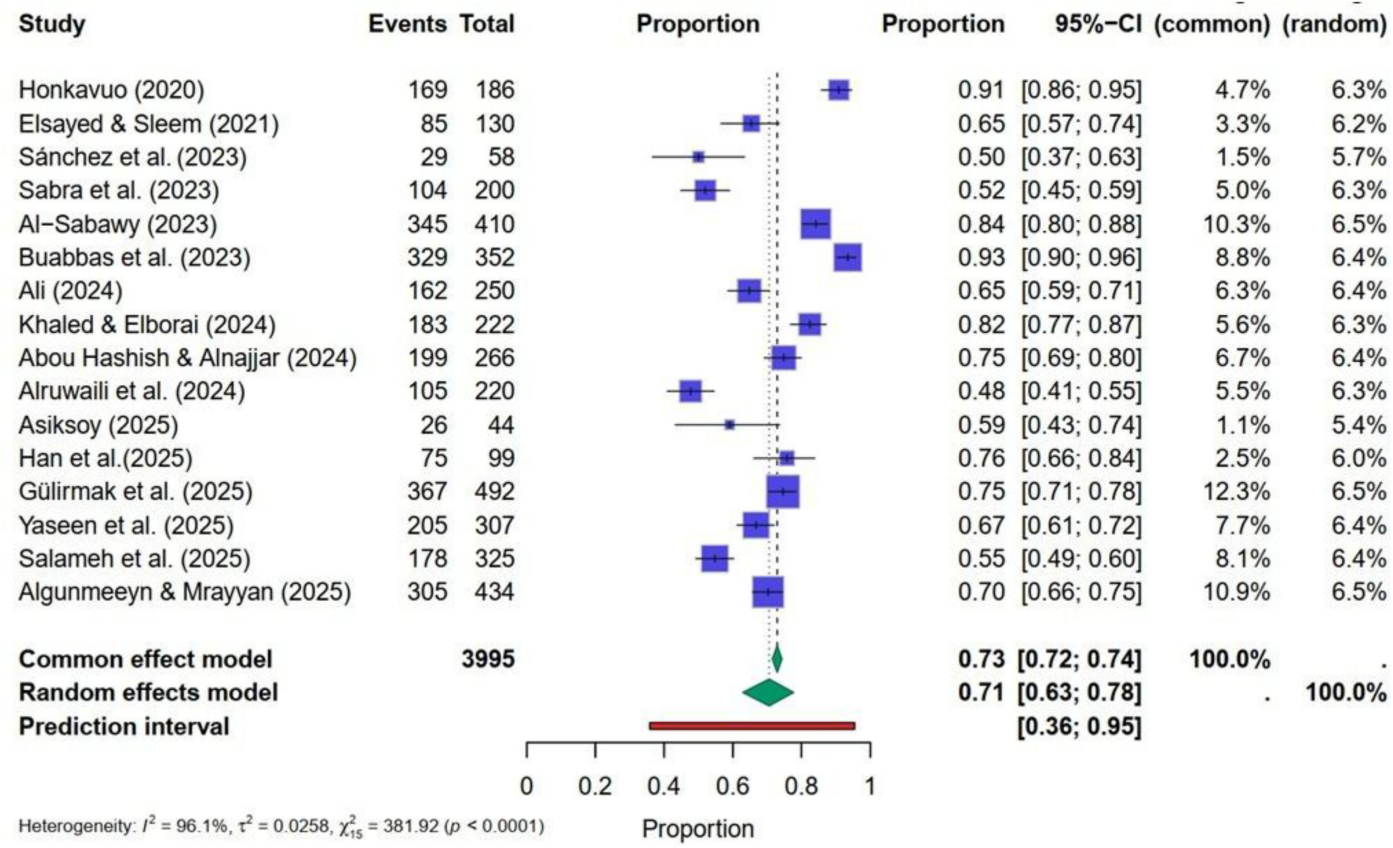
Forest plot for pooled estimates of AI-related attitude in nursing.

Studies related to attitude toward AI also showed very high heterogeneity (Figure 5). Q-statistic = 381.9 (*p* < 0.0001), I^2^ was 96.1%, Tau^2^ = 0.0258. Pooled estimates of the random effect model reported that the average AI-related attitude ranged from 63 to 78%, with a point estimate of 71%. This average is slightly lower than the average AI-related awareness but exhibits a strong level of variation. Overall, findings showed a moderate level of attitude toward AI. Egger’s test yielded an insignificant regression intercept of β_0_ = 0.3115 with *p* = 0.325, which is greater than the 5% level of significance, confirming the absence of strong publication bias in selected studies. Unlike AI-related awareness, meta-regression findings indicate that years of publication were a significant factor in causing variation in AI-related attitudes within the nursing profession. Other characteristics were insignificant for the pooled estimates of AI-related attitude (Table 5). Subgroup analyses revealed a difference in attitudes related to AI between nurses and nursing students. However, this difference was not significant at the 5% level of significance (Table 6). The pooled estimate of nursing students was 74%, which is relatively higher than that of the nursing staff (63%). The level of heterogeneity and variation within the confidence intervals was also high, which necessitated the use of a random effects model. These findings are presented in Table 6.

#### 3.2.3 Findings of meta-analysis for AI-related implementation in nursing-related groups

**FIGURE 6:**
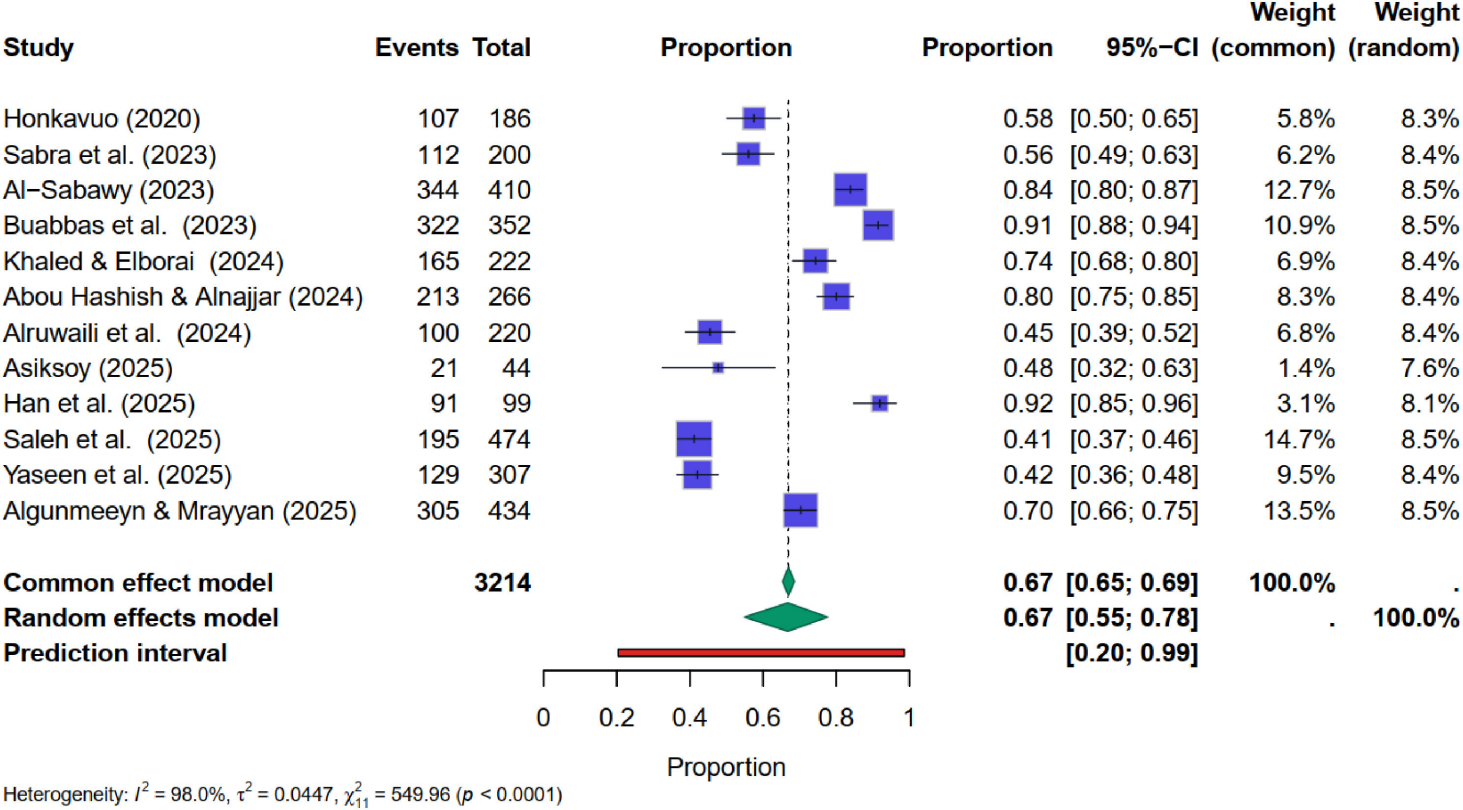
Forest plot for pooled estimates of AI-related implementation in nursing.

According to heterogeneity tests, studies related to AI implementation showed the highest level of variation compared to the knowledge and attitude component of AI (Figure 6). Q-statistic = 549.96 (*p* < 0.0001), I^2^ was 98%, Tau^2^ = 0.0447, and a significant *p*-value indicated the extreme level of heterogeneity. The point estimate of the random effect model provided a 67% AI-related implementation rate with a 95% CI (55–78%). This CI showed a very high level of variation and indicated an acceptable level of AI-related implementation in the nursing profession. Publication bias was not present in the used studies as Egger’s test provided an insignificant regression intercept of β_0_ = −0.342 with a *p* = 0.148, which is greater than the 5% level of significance.

Similar to AI-related awareness, meta-regression findings indicate that no characteristic was a significant contributor to the variation in AI-related implementation percentages (Table 5). Moreover, subgroup analyses revealed an essential difference between nurses and nursing students regarding AI-related implementation. Pooled estimates of nursing students were 78%, which is significantly higher than the nursing staff (55%). These findings are presented in Table 6.

## 4 Discussion

The present study was a unique blend of detailed qualitative and quantitative analysis. Qualitative findings revealed five distinct themes to inform AI-enabled nursing education. Furthermore, quantitative findings indicated moderately high levels of awareness, attitude, and implementation of AI across various nursing groups, but with considerable heterogeneity. Overall, the findings depicted that AI is transforming healthcare globally through the integration of AI in nursing education. AI has a substantial impact on both nursing students and educators; however, a noticeable gap remains between awareness and the practical application of AI. This gap is particularly evident in institutions or countries that have limited resources. Further, little is known about the long-term effectiveness of AI and its transferability across diverse educational contexts.

A substantial body of literature has begun to investigate AI education and training interventions, including workshops, simulation exercises, and online modules. Preliminary findings suggest positive outcomes on AI-related awareness, attitude, and implementation; however, the field lacks a compact and comprehensive synthesis of these efforts. Therefore, this study sought to explore AI integration and its related issues using both qualitative and quantitative methods. Both types of analyses directly and indirectly align with each other. Qualitative synthesis highlights the increased awareness among nursing professionals, which is supported by quantitative findings indicating a high level of awareness. Positive attitudes are reflected in qualitative data, which shows enthusiasm among students. The comparatively weaker implementation of AI in meta-analysis reiterates qualitative findings about infrastructural inequality and a lack of standardized implementation models. Thus, the results offer both confirmation and depth to earlier narrative themes.

Qualitative analysis revealed the foremost issues in AI-enabled nursing education were structural barriers and pedagogical inequalities in accessing AI. The literature review revealed that nursing institutions have begun to integrate AI into their modules or curricula; however, this integration lacks standardization. Different scholarly articles have indicated that many institutions have included AI superficially, highlighting the lack of commitment to curricular reform (19). This poses a serious threat that must be addressed; otherwise, it will lead to significant ethical, social, and legal issues. These disparities in commitment frequently mirror broader geographical and resource-related inequalities. As reflected in Supplementary Appendix A, institutions located in technologically advanced or high-income countries often receive government and policy support for curricular innovation, whereas those in low-resource or developing regions, such as Palestine (57), Egypt (58, 59), and Pakistan (60) struggle with inadequate funding, limited faculty training, and restricted digital infrastructure. Cultural attitudes that prioritize traditional, face-to-face learning may further slow the acceptance of AI-based methods.

The meta-analysis found that the attitudes of nursing faculty and students show an increasing receptiveness to AI. This reflects a cultural shift in health education, viewing AI as a tool for enhancing care, learning, and efficiency. Acceptance of AI, however, was often limited due to ethical issues or structural barriers. Nursing faculty express ambivalence about the use of AI in nursing education due to concerns about potential clinical errors (11). Further, substantial variations were observed in AI-related awareness, attitude, and implementation, which were further augmented with large confidence intervals. The very high heterogeneity (I^2^ > 97%) across pooled analyses indicates a considerable between-study variability, suggesting that the aggregated estimates (awareness: 73%, attitude: 71%, implementation: 67%) should be interpreted with caution. This limitation is acknowledged in the manuscript, which emphasizes that these pooled proportions provide directional rather than definitive evidence. Although substantial variation was observed, the meta-regression analysis (Table 5) did not identify any significant predictors of awareness or implementation, except for the year of publication in the case of attitude (*p* < 0.05). Therefore, these differences should be interpreted cautiously, as the heterogeneity likely reflects unmeasured contextual or methodological influences rather than definitive determinants such as faculty readiness, institutional factors, or resource availability. Part of this heterogeneity also reflects geographic and cultural diversity across the included studies. Institutions from East Asia and Northern Europe, such as South Korea (61) and Finland (62), benefit from long-standing digital literacy initiatives, while Middle Eastern and South Asian contexts, such as Iraq (63), Palestine (57), and Egypt (58), exhibit slower progress due to resource constraints and cautious attitudes toward technology.

Beyond overall variation in the results, subgroup analysis (Table 6) provided more meaningful and stable insights. In comparison to other nursing professionals, nursing students exhibited higher rates of AI-related awareness (79%), positive attitudes (75%), and more frequent implementation (78%). On the other hand, nurses demonstrated significantly lower awareness (66%), attitudes (63%), and especially implantation (56%). These subgroup findings underscore the contextual nature of AI readiness and competency, reflecting real-world educational and professional differences (57). It seems most plausible that nursing students, as novices in the profession, undergo contemporary curricula and are subsequently more attuned to technologies such as AI, which make them better to AI tools. In contrast, working nurses are often unlikely to have received formal training or exposure to AI, which creates a competency gap. This gap underscores the need for immediate action in the form of enhanced educational courses, retraining programs, and organizational policies that incorporate AI frameworks into the career development plans of advanced practice nurses (58, 59). These needs are particularly urgent in low- and middle-income countries where continuing-education opportunities and institutional support remain limited, widening the global competency divide observed in Supplementary Appendix B (57, 58, 64).

In nursing education, the practicality of AI in curricula was the weakest area. A clear gap exists between knowledge and practice, reflecting deeper systemic barriers. The significant challenges included unprepared faculty, limited funding, inadequate digital infrastructure, and a resistant academic community. The study highlighted that tools such as simulation-based learning, AI tutors, and clinical decision aids were rarely accessible outside advanced institutions (32, 54). Thus, many students graduate with an awareness of AI but lack competence. Furthermore, it was noted that demographic disparities affected the outcomes of AI in nursing education. Students in urban, high-income regions had more access to AI tools and modern teaching methods. Those in rural or low-resource settings faced significant access gaps, contributing to a digital divide. As documented in Supplementary Appendix A, countries such as South Korea (65), Finland (62), and the United States (38) show robust institutional and policy support for AI education, whereas settings like Palestine (57), Pakistan (60), Egypt (58), and Iraq (63) report persistent limitations in infrastructure, funding, and curricular flexibility. In several Middle Eastern and Asian studies (57, 63, 64), educators voiced cultural hesitation toward delegating clinical reasoning to AI, revealing how local values and professional norms influence technology adoption. This divide has ethical and professional consequences, potentially exacerbating healthcare inequality (66). These inequalities are not only economic but also cultural, where strong traditions of human-centered care make educators and clinicians more cautious about integrating AI into learning environments (57, 63, 64). Studies showed high heterogeneity, indicative that AI education was fragmented and un-systematized. Implementation depended on institutional leadership, geographic advantage, and faculty readiness each shaped by national resource levels and cultural openness to innovation (57, 58, 62, 65). To summarize, while the direction of nursing education was moving toward AI-enhanced learning, the journey remained fragmented. Standardization, faculty development, equity, and ethics must become central pillars of any future strategy. If unaddressed, the enthusiasm for AI risks being undermined by implementation fatigue and uneven impact.

Overall, these findings emphasize that AI adoption in nursing education follows the contours of geography, economy, and culture. High-income nations with strong digital infrastructures demonstrate smoother integration and better outcomes, whereas resource-constrained or culturally conservative regions progress more slowly. Recognizing these contextual differences is essential for designing globally equitable and culturally sensitive AI curricula.

While the meta-analysis provides valuable insights, several risks and limitations must be acknowledged to contextualize the findings and guide future interpretation. (1) Despite rigorous inclusion criteria, most studies included were from high- and middle-income countries. This geographic imbalance may overrepresent institutions with greater technological resources, leading to skewed conclusions about global readiness. (2) Many studies failed to consistently report demographic details (e.g., gender, age, level of training), limiting subgroup analyses. As a result, it isn’t easy to assess whether AI competency differs significantly across various learner profiles. (3) The nature and intensity of AI-related educational interventions varied widely. Some studies implemented robust simulation training, while others relied on single-session awareness workshops. The pooled effects may therefore dilute or exaggerate the effectiveness of more intensive approaches. (4) Only English-language, peer-reviewed articles were included. This may exclude valuable research published in non-English journals or grey literature, particularly from Global South contexts. Although the overall risk of bias was moderate, the substantial heterogeneity observed among studies (I^2^ > 97%) suggests that factors beyond bias, such as variability in methodology and populations, may have influenced the pooled estimates. Despite these risks, the combination of sensitivity analysis, model triangulation, and qualitative triangulation strengthens the reliability of core findings.

## 5 Conclusion

Thematic analysis identified the need for standardized AI education, targeted faculty development, infrastructural support, and equitable access to digital tools. If these areas are not addressed, nursing education may face challenges in keeping pace with technological progress and in preparing graduates for leadership within AI-enabled healthcare systems. This gap highlights the need to enhance institutional readiness and policy support, ensuring that growing individual enthusiasm for AI is effectively translated into sustainable education and professional development within nursing. Furthermore, a meta-analysis substantiated the growing interest in and partial implementation of AI literacy and competency in nursing education globally. Awareness and attitudes were encouragingly high, but practical usage and skill-based applications remained inconsistent. Regional disparities, generational gaps, and institutional readiness significantly shaped the variation in outcomes. Analyses also indicated an alarming level of inconsistency, combined with high levels of heterogeneity across studies, raising essential debates about achieving an ideal balance and preparedness among diverse nursing populations. Overall, the findings suggest a gradual but uneven global shift toward AI integration in nursing education, reflecting both opportunities and persistent disparities. In conclusion, this review offers evidence-informed recommendations for educators, institutions, and policymakers to strengthen standardization, capacity-building, and equitable access so that nursing education remains inclusive and future-ready.

## 6 Recommendations

To ensure ethical, inclusive, and impactful integration of AI in nursing education, several key recommendations are proposed. National-level nursing councils and health ministries should mandate the inclusion of core AI competencies in both undergraduate and graduate nursing curricula. Simultaneously, there must be substantial investment in faculty development programs that integrate digital literacy, ethical considerations, and instructional design. Bridging the digital divide is equally critical and can be achieved by funding simulation labs, providing access to AI software, and improving internet connectivity in under-resourced areas. Furthermore, AI educational content should be tailored to address regional health priorities, local data infrastructures, and cultural contexts to enhance relevance and applicability. Lastly, ongoing research is crucial for evaluating the long-term effects of AI education on clinical decision-making and patient outcomes. Collectively, these actions will empower future nurses to utilize emerging technologies effectively and lead in an AI-enhanced healthcare environment.

## Data Availability

All data supporting the findings of this study are included within the article and its Supplementary Files. No additional datasets were generated or analyzed during the current study. Requests for further information may be directed to the corresponding author at waqas@qu.edu.qa.
