# Supplementary material for: AI literacy and competency in nursing education: preparing students and faculty members for an AI-enabled future-a systematic review and meta-analysis
Source: Front Med (Lausanne). 2025 Nov 26;12:1681784. doi: 10.3389/fmed.2025.1681784 (PMC12689331; doi:10.3389/fmed.2025.1681784)
Supplement: Supplementary file 1 [file Table_1.docx]

**Appendix A: Summary of Included Studies in the Systematic Review and Meta-Analysis**

| **S. No.** | **Authors and Year** | **Research Design** | **Study Aim** | **Sample** | **Setting** | **Findings/Conclusions** |
| --- | --- | --- | --- | --- | --- | --- |
|  | Makhlouf et al., 2024 | Quasi-experimental design | To evaluate the effectiveness of a knowledge-based AI chatbot integrated into a nursing training program in enhancing students’ knowledge and engagement | 120 nursing students (60 experimental, 60 control) | University-based nursing program | AI chatbot significantly improved students' knowledge scores and engagement levels in the experimental group compared to the control group, indicating its effectiveness as a supplementary educational tool in nursing education(73). |
|  | White et al., 2024 | Mixed-method design | To explore the impact of an AI-based virtual simulation on nursing students’ attitudes toward older adults | 85 undergraduate nursing students | Nursing school/university simulation lab | The AI virtual simulation improved students' empathy and attitudes toward older adults. Qualitative findings revealed increased awareness, emotional connection, and appreciation for geriatric care(74). |
|  | Salama et al., 2025 | Cross-sectional descriptive study | To assess the knowledge, attitudes, and practices of nursing students regarding the use of AI technology, specifically ChatGPT | 543 nursing students from various Palestinian universities | Palestinian universities | Most students had limited knowledge but showed positive attitudes toward AI technologies like ChatGPT. However, practical use was minimal, indicating a need for curricular integration and digital literacy enhancement(75). |
|  | Labrague et al., 2025. | Scoping Review | To explore and categorize AI-based teaching strategies in nursing education and examine their effectiveness, applications, and outcomes | 16 research articles | Not applicable (literature review) | AI teaching strategies were classified into three categories: AI-driven simulations, AI-augmented instruction, and AI-generated content. These strategies enhanced student engagement, clinical understanding, critical thinking, and preparation. The study suggests integrating AI into nursing curricula and calls for further research on long-term impacts(8). |
|  | Kahraman et al., 2025 | Descriptive cross-sectional | Determine AI literacy levels among perioperative nurses | Perioperative nurses | Türkiye | Moderate AI literacy levels; usage influenced by exposure and training(76) |
|  | Jallad et al., 2024. | Cross-sectional quantitative study | To explore the prevalence of AI tool usage in nursing education and identify factors associated with their adoption | 578 nursing students and faculty members | Nursing colleges across multiple institutions | The study revealed a moderate incidence of AI tool usage in nursing education. Key factors influencing adoption included age, technological proficiency, faculty support, and institutional resources. The authors recommend structured training and infrastructure development to support AI integration(77). |
|  | Ramadan et al., 2024. | Qualitative study | Explore nurses' perspectives on AI adoption | 48 Registered nurses | Saudi Arabia | Identified facilitators (e.g., perceived usefulness) and barriers (e.g., lack of training) to AI adoption(31). |
|  | Almagharbeh et al., 2025. | Qualitative study (descriptive phenomenological approach) | To explore Jordanian nurses’ perceptions, experiences, and attitudes regarding the use of artificial intelligence in clinical nursing practice | 18 registered nurses | Public and private hospitals in Jordan | Nurses recognized AI's potential to enhance efficiency, reduce workload, and support decision-making. However, they expressed concerns about ethical issues, data privacy, lack of training, and fear of professional replacement. The study recommends policy development, ethical guidelines, and comprehensive training for effective AI integration(78). |
|  | 1. K. M. El-Sayed et al., 2025 | Cross-sectional | Examine the impact of AI literacy and innovation mindset on self-efficacy | 596 nursing students | Alexandria University, Egypt | AI literacy and innovation mindset positively influenced career and talent self-efficacy(10) |
|  | Migdadi et al., 2024. | Cross-sectional correlational study | To investigate how AI ethical awareness, attitudes, and anxiety relate to nursing students' intention to use AI technologies | 812 nursing students | Nursing faculties in Jordan | Ethical awareness and positive attitudes significantly predicted intention to use AI, while anxiety negatively influenced it. The findings emphasize the importance of fostering ethical understanding and reducing anxiety through targeted training and curriculum integration to improve AI adoption in nursing education(79). |
|  | Kwan et al., 2025. | Conceptual / Perspective Review | To explore the opportunities, challenges, and required strategic actions for integrating AI into nursing practice, education, and research | Not applicable | Global / Multidisciplinary nursing perspective | AI presents transformative potential in nursing education, research, and practice by enhancing learning, clinical decisions, and research capacities. Key challenges include lack of AI training in curricula, risks of dehumanization, and ethical concerns (e.g., data privacy, bias). Strategic actions proposed include revising core competencies, integrating ethics, and reinforcing humanistic values(80). |
|  | Srinivasan et al., 2024. | Perspective / Viewpoint Article | To explore the pedagogical implications, benefits, and risks of integrating AI and chatbots in nursing education | Not applicable | Global / Academic perspective | The article emphasizes that AI and chatbots can enhance nursing education by enabling personalized, scalable, and interactive learning. However, it also raises concerns regarding ethical implications, over-reliance, and the need for proper faculty training. The authors call for thoughtful, value-driven integration aligned with educational goals(23). |
|  | Rajah et al., 2025. | Qualitative narrative inquiry | To explore the experiences and perceptions of nursing students at Bayero University, Kano, regarding the utilization of artificial intelligence (AI) in their academic and clinical environments. | 20 Nursing students at Bayero University, Kano (exact number not specified) | Department of Nursing, Bayero University, Kano, Nigeria | Most students used AI for academic tasks but lacked awareness of nursing-specific tools; despite tech challenges, they viewed AI positively while voicing concerns about over-reliance and job loss, highlighting the need for better infrastructure and targeted training(81). |
|  | Labrague, Aguilar-Rosales, Yboa, & Sabio, 20223. | Cross-sectional study | To examine student nurses' readiness to embrace AI technology, explore associated factors, and identify perceived barriers to accessing AI technology. | 323 student nurses from a public nursing school in the Philippines | Public nursing school in the Philippines | Student nurses showed a moderate readiness to embrace AI in their studies, while also perceiving notable barriers to its access. Key factors influencing readiness included technological proficiency, understanding of AI, and perceived relevance to nursing practice. Reported barriers included limited computer skills, lack of AI awareness, and time constraints. The study recommends improving digital literacy, increasing AI-related knowledge, and offering hands-on experiences to better prepare nurses for an AI-integrated healthcare system(58). |
|  | Abdelaziz et al., 2025. | Literature review | To investigate existing literature on registered nurses' and nursing students' attitudes toward advanced technology and artificial intelligence in nursing, including nursing education. | Ten peer-reviewed studies published between 2017 and 2022 | Various clinical and educational settings across the United States | Nursing students expressed positive perceptions of AI, such as enhanced patient care, greater efficiency, and reduced errors. However, concerns included job displacement, diminished human interaction, and ethical or legal implications. Challenges highlighted involved implementation barriers, insufficient training, and the risk of reduced human connection in care. Understanding these perceptions is crucial for the ethical and effective integration of AI into nursing education and practice(82). |
|  | 1. J. Hwang et al., 2022. | Systematic review | To analyze research trends in artificial intelligence (AI) applications within nursing activities from 2001 to 2020, focusing on dimensions such as nursing activities, research samples, methods, AI roles, algorithms, evaluation measures, and research foci. | 102 peer-reviewed studies published between 2001 and 2020 | Studies sourced from the Web of Science database | The primary roles of AI identified were profiling and prediction, followed by assessment and evaluation. The main research focus was on the design or evaluation of AI systems/instruments, with secondary attention to correlation and affective issues. Recommendations include expanding research into diverse nursing activities, employing varied research methods, and addressing ethical considerations in AI applications(83). |
|  | Labrague & A1 Harrasi, 2025. | Systematic Review | To assess nursing students' perceptions of AI through the Technology Acceptance Model (83), focusing on perceived usefulness, ease of use, attitude, and intention to use | 15 studies included various samples of nursing students across reviewed literature | Academic nursing education settings across multiple countries (via included studies) | Positive attitudes toward AI are shaped by its perceived usefulness and ease of use. These factors significantly influence students’ intention to adopt AI. Recommendations include improving AI tool usability and showcasing its educational benefits to foster better adoption in nursing education(85). |
|  | Shang, 2021. | Concept Analysis (Walker and Avant’s method) | To clarify the meaning, attributes, antecedents, and consequences of using Artificial Intelligence (AI) in nursing practice. | Not applicable (conceptual analysis) | Theoretical framework in nursing context | Defined key attributes of AI and its application in nursing(86). |
|  | Prasad et al., 2023. | Multidimensional Evaluation Study | To assess the applicability, effectiveness, and challenges of using generative AI models in both theoretical and clinical nursing education. | Not explicitly stated (evaluation-based discussion) | Theoretical and clinical nursing education contexts | Suggested AI enhances teaching strategies and content accessibility(68). |
|  | Albikawi & Abuadas, 2025. | Scale Development and Validation Study | To develop and validate a novel scale to measure nursing students’ fear of artificial intelligence. | Nursing students (exact number not specified) | Likely university or nursing education programs | Developed a validated scale highlighting anxiety about AI integration(67). |
|  | Huang et al., 2021. | Reflective/Discussion Paper | To reflect on nursing education and evaluate how to ensure the nursing profession is prepared for the age of AI. | Not applicable (conceptual/reflection) | Nursing education and professional development context | Urges proactive curriculum reform for AI readiness(87). |
|  | Suwalka et al., 2024. | Conference Paper / Exploratory Study | To explore the integration of AI applications and simulation-based learning as the future of nursing education. | Not explicitly stated | Nursing education, AI, and simulation-based learning environments | Found AI simulates real-life scenarios improving critical thinking(88). |
|  | Hyun & Ran, 2024. | Trend Analysis / Bibliometric Study | To analyze recent research trends related to artificial intelligence in the field of nursing. | Published nursing AI-related research articles | Academic literature in nursing and AI domains | AI research has shifted from theory to practical application(89). |
|  | G.-J. Hwang et al., 2022. | Bibliometric and Content Analysis | To profile the roles, applications, and trends of AI in nursing education research from 1993 to 2020. | 107 journal articles on AI in nursing education | Global research publications in nursing education | AI has diversified, but ethical oversight is lagging(90). |
|  | Seibert et al., 2021. | Rapid Review | To identify and analyze application scenarios for AI in nursing care. | 46 relevant studies on AI in nursing care | Nursing care settings and healthcare contexts | AI supports decision-making but needs regulatory clarity(91). |
|  | Martinez-Ortigosa et al., 2023. | Systematic Review | To examine the current applications of artificial intelligence in nursing care. | 30 selected studies on AI in nursing care | Global nursing care and clinical settings | Found AI supports monitoring, diagnosis, and patient communication(92) |
|  | Asiri et al., 2024. | Literature Review | To explore how AI is revolutionizing patient diagnosis and treatment strategies in nursing care. | Recent literature on AI in nursing care | Healthcare and clinical nursing environments | AI is revolutionizing care but implementation is inconsistent(93). |
|  | Alsaeed et al. | Conceptual/Review-Based Study | To examine the role of AI and predictive analytics in enhancing decision-making in nursing through health IT. | Not explicitly stated (review-based) | Nursing care, health information technology context | Highlights AI’s role in early detection and resource optimization(94). |
|  | Fengxia & Dan. | Discussion/Analytical Paper | To explore the challenges, opportunities, and developmental pathways for integrating AI into pediatric nursing education. | Not applicable (conceptual analysis) | Pediatric nursing education context | AI has potential for individualized learning but requires policy support(95). |
|  | Nassef & Zeid, 2024. | Descriptive Literature Review | To review and describe the available evidence on the use and impact of artificial intelligence in nursing education. | Published literature on AI in nursing education | Academic and educational nursing settings | Need for structured AI education strategies in curricula(96). |
|  | Patel, 2025 | Narrative Review | To review the implementation and impact of artificial intelligence in nursing education. | Not explicitly stated (review-based) | Nursing education and training environments | Found improved engagement, with digital divides as a barrier(97). |
|  | Jiang, 2024. | Qualitative/Contextual Review | To explore the challenges and opportunities of integrating AI into nursing education within the Chinese context. | Not explicitly stated | Nursing education in China | Challenges include resource inequality and faculty training(98). |
|  | Glauberman et al., 2023 | Discussion/Analytical Review | To examine the opportunities and challenges of implementing artificial intelligence in nursing education. | Not explicitly stated | Nursing education and training settings | Need for educator support and ethical guardrails(99). |
|  | Tushe, 2025. | Comprehensive Literature Review | To provide an overview of current applications and future prospects of AI in nursing practice and education. | Not explicitly stated | Nursing education and clinical practice settings | AI’s success depends on collaboration and ethics training(69). |
|  | Hassanein et al., 2025. | Integrative Review | To examine the clinical and operational impacts of artificial intelligence in nursing practice. | Studies from diverse clinical settings | Clinical nursing and healthcare operations | Demonstrates increased efficiency but requires training(100). |
|  | Adomat, 2024. | Narrative Review / Theoretical Analysis | To explore current applications, ethical considerations, and future prospects of AI in nursing. | Not explicitly stated | Nursing practice and ethical discourse | Encourages ethics-focused training and regulation(101). |
|  | Ramirez-Baraldes et al., 2025. | Commentary / Analytical Review | To discuss the new opportunities and challenges posed by AI in nursing from an educational perspective. | Not explicitly stated | European nursing education and practice | AI use must align with patient-centered care(102). |
|  | Kimiafar et al., 2023. | Systematic Review | To assess the level of AI literacy among healthcare professionals and students. | Studies assessing AI literacy | Healthcare and academic settings | Literacy is low; formal education needed urgently(103). |
|  | Hong et al., 2024. | Scoping Review | To map and explore the scope of research on artificial intelligence applications in nursing. | Research articles (scoping scope) | Nursing education and clinical practice | Growth in AI research, but mostly conceptual at present(104). |
|  | Stamer et al., 2023. | Scoping Review | To examine how artificial intelligence supports the training of communication skills in health professions education. | Research articles involving AI and communication training | Health care professional education settings | Found improved communication through AI-driven training(84). |
|  | Salimiet al., 2025. | Narrative Review | To review the development and integration of AI-based nursing curricula in Iran and other Gulf countries. | Not explicitly stated | Nursing education in Iran and Gulf countries | Curricula are emerging; standardization is lacking(105). |
|  | Von Gerich et al., 2022. | Scoping Literature Review | To synthesize current evidence on AI-based technologies used in nursing practice and education. | 93 included studies | Global nursing practice and education settings | AI is promising but ethical, educational frameworks are needed(106). |
|  | Yeung et al., 2025. | Scoping Review | To examine the use of AI-based technologies in communication training within nursing education. | Research studies on AI in communication training | Nursing education and simulation-based training | AI supports simulation-based communication learning(107). |
|  | Naureen et al., 2025. | Descriptive Cross-Sectional Study | To assess awareness of the role of AI in healthcare among undergraduate nursing students. | 162 undergraduate nursing students (Pakistan) | Academic nursing education environment | Awareness moderate; need for AI integration in curriculum(108). |
|  | Sampayan, 2025. | Analytical/Descriptive Study | To explore the challenges and future prospects of using Blackboard LMS as an AI-supported tool in nursing education. | Not explicitly stated | Nursing education using Blackboard LMS | AI enhances LMS functionality but raises concerns over personalization(109). |
|  | Couper, 2024. | Commentary/Analytical Perspective | To discuss the key challenges and emerging opportunities associated with AI in nursing education. | Not explicitly stated | Nursing education and training contexts | Opportunities abound but require institutional readiness(53). |
|  | Sukmawati et al., 2025. | Systematic Literature Review | To examine the use of gamification and AI in English language training for nurses and its outcomes. | Not explicitly stated | Nursing language education and professional training | Gamified AI tools improve engagement and learning outcomes(110). |
|  | Richard G. Booth et al., 2021. | Expert Commentary / Perspective | To outline how the nursing profession should evolve and adapt for a digital and AI-driven future. | Not applicable | Nursing education, clinical practice, and policy | Advocates proactive policy-making and digital upskilling(6). |
|  | Kouka et al., 2025. | Narrative Review | To explore how artificial intelligence is being implemented in nursing education and its implications. | 7 articles were taken in final analysis | Nursing education systems and curricula | Emphasizes the need for faculty development and policy(111). |
|  | Yasin et al., 2025. | Scoping Review | To explore the incorporation of artificial intelligence into nursing research, identifying key areas and trends. | 20 Studies involving AI in nursing research | Nursing research and academic settings | Research is expanding, but still fragmented(112) |
|  | Chan et al., 2024. | Scoping Review | To examine how artificial intelligence is being integrated into nursing simulation education. | 14 Studies on AI in simulation education | Nursing simulation and clinical training settings | Supports AI as an effective tool in high-fidelity learning(113). |
|  | Tenorio et al., 2023. | Literature Review / Conference Paper | To review competence areas, pedagogical approaches, contexts, and formats for introducing AI literacy in schools. | 31 cases of educational frameworks and programs | General education (AI literacy focus, not nursing-specific) | Frameworks must evolve to prepare future professionals(114). |
|  | Borromeo et al., 2025. | Bibliometric Review | To analyze global research trends on the use of generative AI in nursing education. | Published literature on generative AI in nursing | Nursing education and academic research settings | Revealed rapid growth and thematic clustering in AI use(115). |
|  | Lora & Foran, 2024. | Integrative Review | To explore nurses' perceptions of AI integration into clinical practice. | Studies on nurses’ perspectives | Perioperative and general nursing practice | Found cautious optimism but need for support(116). |
|  | Carrington et al., 2024. | Book Chapter / Conceptual Discussion | To discuss the integration of artificial intelligence into nursing education through applied informatics. | Not explicitly stated | Nursing education and health informatics | Emphasizes the need for updated curricula and digital competencies in nursing education(116). |
|  | Ostick et al., 2025. | Integrative Review | To explore nursing students’ and faculty members’ attitudes, perceptions, and behavioral intentions toward AI use in nursing education. | 6 Studies focused on nursing education stakeholders | Nursing academic and training environments | Positive attitude toward AI with concerns about data privacy and the need for training(117). |
|  | Moustaq Karim Khan Rony et al., 2025. | Qualitative Descriptive Study | To explore nursing students’ perspectives on integrating AI into clinical practice and training. | 25 Nursing students | Clinical training and nursing education settings | Emphasizes evidence-backed integration and policy changes(118). |
|  | Turchioe et al., 2023. | Survey-Based Quantitative Study | To identify opportunities and challenges in using digital health and AI to support nurses, based on informaticists’ perspectives. | 52 Nursing informaticists | Digital health and informatics in nursing practice | Identified need for AI-specific competencies and institutional investment(119). |
|  | Buchanan et al., 2021. | Scoping Review | To explore predicted influences of artificial intelligence on nursing education. | 27 Studies on AI and nursing education | Nursing education systems and academic programs | AI is anticipated to personalize learning and enhance decision-making support(15). |
|  | Shen et al., 2025. | Qualitative Study | To explore nursing students’ experiences and concerns related to integrating AI—particularly prompts, privacy, and personalized learning—into education. | Nursing students | AI-integrated nursing education environments | Emphasized ethical concerns and the need for student-centered AI design(18). |
|  | Wu, 2024. | Theoretical/Conceptual Paper | To discuss how AI innovations are transforming and potentially subverting traditional nursing education models. | Not explicitly stated | Nursing education and pedagogical innovation | AI disrupts traditional roles; educators must be prepared to adapt(120). |
|  | Othman et al., 2025. | Conceptual/Review Paper | To examine the evolving role of nursing informatics within the context of artificial intelligence. | Not explicitly stated | Nursing informatics in healthcare and education | Nursing informatics is becoming increasingly central to integrating AI in clinical practice, emphasizing the need for upskilling and system-level readiness(121). |
|  | Lin et al., 2024. | Conceptual/Strategic Framework | To identify key elements and implementation strategies for integrating AI into future nursing education. | Not explicitly stated | Nursing education and curriculum development | The study emphasizes curriculum redesign, faculty training, and infrastructure development as essential strategies for successful AI integration in nursing education(122). |
|  | Qtait, 2025. | Systematic Review | To assess the impact of artificial intelligence on nursing students' learning, skills, and perceptions. | Studies involving nursing students and AI | Nursing education and academic environments | Found enhanced engagement, motivation, and digital readiness among students(123). |
|  | Wei et al., 2025. | Integrative/Comprehensive Review | To examine current applications, challenges, and future directions of AI integration in nursing. | Not explicitly stated | Clinical nursing practice and education | Calls for balancing innovation with ethical considerations and usability(124). |
|  | Luo et al., 2024. | Bibliometric Analysis | To identify research hotspots and theme trends in AI-related nurse education from 1994 to 2023. | 135 Publications from bibliographic databases | Nurse education research landscape | Shows rapid growth in AI research, with focus shifting toward pedagogy(125). |
|  | El Arab, A1 Moosa, et al., 20205. | Umbrella Review | To synthesize findings from existing reviews on the role of AI in nursing education and clinical practice. | 18 Systematic and scoping reviews | Nursing education and clinical practice settings | Evidence shows AI improves efficiency, but gaps remain in ethical frameworks(126). |
|  | 1. K. K. Rony et al., 2025. | Umbrella Review | To synthesize evidence from existing reviews on the applications and impacts of AI in nursing care. | 13 Published reviews on AI in nursing care | Clinical nursing and healthcare environments | AI aids in care delivery but requires nurse training and policy development(127). |
|  | Ma et al., 2025. | Systematic Review | To comprehensively assess how artificial intelligence is influencing and transforming nursing education. | 15 Empirical studies on AI in nursing education | Nursing education across academic settings | Highlights AI’s role in personalized learning and competence assessment(128). |
|  | Lane et al., 2024. | Theoretical/Discussion Paper | To explore the dual role of generative AI as both a helpful tool and a potential threat in nursing education, and propose guidance strategies. | Not explicitly stated | Nursing education and academic environments | Advocates for guided, ethical AI use with educator involvement(129). |
|  | Alenazi & A1-Anazi, 2025. | Descriptive Qualitative Study | To explore nursing students’ understanding and perceptions of artificial intelligence. | 20 Nursing students | Nursing education programs in Saudi Arabia | Found positive perceptions, but stress need for AI literacy(27). |
|  | Harmon et al., 2021. | Scoping Review | To explore the use of AI and virtual reality in clinical simulation for nursing pain education. | Studies involving AI/VR in simulation | Clinical simulation in nursing education | Improved student understanding of pain assessment(130). |
|  | Seo & Kim, 2024. | Topic Modeling / Content Analysis | To analyze research trends on generative AI in nursing education using topic modeling techniques. | 139 Scholarly articles on AI in nursing education | Academic and educational research context | Identified themes include chatbot use and competency development(131). |
|  | De Gagne, 2023. | Educational Intervention / Conceptual Paper | To explore how values clarification exercises can help prepare nursing students for AI integration. | Nursing students | Nursing education and training environments | Values-based training helps mitigate AI risks(25). |
|  | Mina, 2024. | Descriptive/Practice-Based Report | To propose a holistic approach for enhancing clinical instructors’ preparedness in integrating AI into nursing education. | Clinical instructors (target audience) | Nursing education and clinical instruction | Holistic instructor readiness is key to effective integration(132). |
|  | Schneidereith & Thibault, 2023. | Narrative/Descriptive Review | To explain AI fundamentals and provide recommendations for nurse educators. | Not applicable | Nursing education context | Emphasizes foundational AI literacy and advocates faculty training for effective integration(133). |
|  | O’Connor et al., 2023. | Systematic Review | To examine the use, benefits, challenges, and ethics of AI in nursing and midwifery. | 140 studies | Nursing and midwifery practice settings | AI enhances workflow, documentation, and clinical accuracy(134). |
|  | Abuzaid et al., 2022. | Exploratory cross-sectional survey | To assess knowledge, attitudes, willingness, and organizational readiness for integrating AI into nursing practice. | 200 registered nurses | Health organizations in the United Arab Emirates | Nurses showed moderate readiness and positive attitudes but highlighted institutional and training gaps(29). |
|  | Amin et al., 2025. | Mixed-method study | To explore nurses' sentiments toward AI and resistance to change in healthcare organizations. | 500 nurses (quantitative), 17 nurses (qualitative) | Various healthcare organizations | Resistance stemmed from a lack of awareness and training; qualitative data emphasized the role of leadership(135). |
|  | 1. J. Labrague, Aguilar-Rosales, Yboa, and Sabio 2023. | Cross-sectional study | To examine student nurses' readiness to embrace AI technology, explore associated factors, and identify perceived barriers to accessing AI technology. | 323 student nurses | One public-owned nursing school in the Philippines | Students were moderately ready for AI but lacked access, training, and institutional support(57). |
|  | Labrague, Aguilar-Rosales, Yboa, Sabio, & Santos, 2023. | Cross-sectional study | To investigate the attitudes and intentions of student nurses towards AI in nursing practice and explore the relationship between their attitudes, perceptions of AI utilization, and intentions to adopt AI technology. | Not specified | Not specified | Positive attitudes correlated with higher intention to use AI, highlighting importance of perceptions(136). |
|  | Lukic et al., 2023. | Cross-sectional multi-center study | To assess first-year nursing students' attitudes towards artificial intelligence in nursing. | Not specified | Multiple centers | Found cautious optimism; students acknowledged AI’s relevance but feared replacement and job changes(137). |
|  | Mariano et al., 2025. | Cross-sectional analysis | To assess the knowledge, attitudes, and practices of nurses, nursing faculty, and students regarding AI in Saudi Arabia. | Not specified | Saudi Arabia | Participants demonstrated moderate knowledge and generally positive attitudes; suggested inclusion of AI in nursing curricula(12). |
|  | Xuto et al., 2025. | Validation study | To evaluate the reliability, accuracy, precision, and external validity of an AI-assisted answer assessment (4A) program for cognitive assessments in nursing education. | 170 nursing students from northern Thailand (52 randomly selected for detailed testing) | Northern Thailand | The AI-based 4A program showed high reliability and validity, indicating strong potential for use in cognitive assessments(138). |
|  | Yalcinkaya et al., 2024 | Cross-sectional study | To explore nursing students' attitudes and readiness for artificial intelligence in nursing education. | Not specified | Not specified | Found generally positive attitudes; students expressed a need for AI training and integration in coursework(24). |
|  | Yang, 2024. | Correlational study | To examine the influences of digital literacy and moral sensitivity on AI ethics awareness among nursing students. | 140 nursing students from universities in G City and J Province, South Korea | South Korea | Higher digital literacy and moral sensitivity significantly predicted increased AI ethics awareness(139). |
|  | Shin et al., 2023. | Quasi-experimental study | To evaluate the effects of AI-assisted learning on nursing students' ethical decision-making and clinical reasoning in pediatric care. | 99 nursing students enrolled in a pediatric nursing course, randomly divided into an experimental group (using ChatGPT) and a control group (using traditional textbooks). | Educational setting involving a pediatric nursing course. | The experimental group using ChatGPT showed significantly better ethical decision-making and clinical reasoning than the control group(23). |
|  | 1. A. I. El-Sayed et al., 2025. | Cross-sectional study | To examine the relationship between evidence-based practice (EBP) climate, AI competence self-efficacy, and creativity-nurturing behaviors among nurse educators. | 570 Nurse educated | Various educational institutions | Positive correlations were found among EBP climate, AI competence self-efficacy, and creativity-nurturing behaviors(140). |
|  | 1. K. M. El-Sayed et al., 2025. | Cross-sectional study | To assess how AI literacy and innovation mindset influence nursing students' career and talent self-efficacy. | Not specified | Higher education institutions in Egypt | AI literacy and innovation mindset were significant predictors of career and talent self-efficacy(10). |
|  | 1. Han et al., 2025. | Cross-sectional online survey | To explore nursing students' use and perceptions of generative AI in nursing education. | Not specified | Various educational institutions | Students reported frequent use of generative AI and held positive perceptions, but highlighted concerns about ethics and academic integrity(70). |
|  | Qutishat et al., 2025. | Scoping review | To identify benefits, challenges, and future recommendations for integrating AI into nursing education. | Not applicable | Teaching and Learning in Nursing journal | Benefits include personalized learning and administrative support; challenges involve lack of training, ethical issues, and resistance to change; recommendations highlight curriculum reform and faculty development(141). |
|  | Ostick et al., 2025. | Systematic review | To synthesize current evidence on the impact of AI on nursing students' education, experiences, and preparedness. | Not applicable | Various studies | The review found that AI enhances student learning, engagement, and preparedness but raises concerns about ethics, bias, and the need for faculty support(117). |
|  | Dehghani et al., 2025. | Systematic review | To identify the main challenges in implementing artificial intelligence in nursing education. | 18 articles included in this study | Educational Research in Medical Sciences | Key challenges identified include lack of faculty training, infrastructural deficits, ethical dilemmas, and curriculum gaps; emphasized need for strategic planning and policy support(142). |
