# Supplementary material for: AI literacy and competency in nursing education: preparing students and faculty members for an AI-enabled future-a systematic review and meta-analysis
Source: Front Med (Lausanne). 2025 Nov 26;12:1681784. doi: 10.3389/fmed.2025.1681784 (PMC12689331; doi:10.3389/fmed.2025.1681784)
Supplement: Supplementary file 2 [file Table_2.docx]

**Appendix B: Basic Characteristics of Quantitative Studies**

| **Sr. No.** | **Authors and Year** | **Research Design** | **Study Aim** | **Sample** | **Setting** |
| --- | --- | --- | --- | --- | --- |
|  | Abou Hashish and Alnajjar 2024 | Cross-sectional survey | To assess nursing students' perceived knowledge, attitudes, and skills regarding digital transformation, digital health literacy, and attitudes toward AI. | 266 nursing students | Universities in Egypt and Saudi Arabia(27) |
|  | Algunmeeyn and Mrayyan 2025 | Cross-sectional online survey | To explore nursing students' perceptions of AI use in nursing research. | 434 nursing students | Online survey across various institutions(62) |
|  | Ali 2024 | Descriptive research design | To assess challenges and barriers of using AI as perceived by nursing personnel. | 250 nursing personnel | EL Fayoum University hospitals, Egypt(60) |
|  | Al-Sabawy 2023 | Cross-sectional exploratory survey | To explore nurses' attitudes and perceptions toward the implementation of AI in nursing practice. | 410 nurses | Kirkuk Health Department, Iraq(65) |
|  | Gulsum Asiksoy 2025 | Mixed-methods phenomenological study | To evaluate the medical reliability and patient safety implications of AI-powered chatbots in health literacy education. | 44 nurses | Private hospital in Cyprus(144) |
|  | Khaled and Elborai 2024 | Descriptive cross-sectional study | To assess nursing students' knowledge and attitudes regarding AI. | 222 | Nursing faculties in Egypt(61) |
|  | Qaladi et al. 2025a | Cross-sectional survey | To explore registered nurses' perceptions of AI in Saudi Arabia, focusing on both challenges and opportunities. | 202 nurses | Saudi Arabia(70) |
|  | Sabra et al. 2023 | Descriptive research design | To assess nurses' perspectives and attitudes toward AI utilization in healthcare. | 200 nurses | Qena University Hospitals(145) |
|  | Salameh et al. 2025 | Cross-sectional study | To explore nursing students' attitudes toward AI in Palestine. | 325 | Palestine(59) |
|  | Yaseen et al. 2025 | Descriptive cross-sectional study | To assess nurses' knowledge regarding the application of AI in nursing practice. | 307 | Not specified(146) |
|  | Buabbas et al. 2023 | Cross-Sectional Study | Assess medical students’ perceptions and attitudes towards AI in healthcare. | 352 nursing students | Kuwait University(147) |
|  | Gülırmak Güler and Şen Atasayar (2025) | Descriptive and Relational Study | To examine the relationship between nursing students' attitudes toward AI and their creative personality traits. | 492 nursing students from a Turkish university | Nursing programs in Turkey(148) |
|  | J.-w. Han et al. 2025 | Quasi-Experimental / Program Evaluation | To develop and test a chatbot-based education program supporting self-directed learning in nursing students. | 99 nursing students | South Korean nursing program (63) |
|  | Elsayed and Sleem 2021 | Descriptive research design | To investigate nurse managers' perspectives and attitudes toward using AI technology in health settings. | 130 nurse managers | Mansoura University Hospitals, Egypt(149) |
|  | Honkavuo 2020 | Quantitative descriptive study | To assess nursing students' views on digital teaching methods in nursing education. | 186 | Nursing schools in Finland(64) |
|  | Sánchez and Karaksha (2023) | Quantitative, non-experimental, descriptive, and exploratory study | To determine the attitudes of undergraduate nursing students toward e-learning. | 71 sophomore nursing students (58 responded; 82.8% female) | Not specified(150) |
|  | Saleh et al. 2025 | Cross-sectional study | To examine faculty perceptions of artificial intelligence (AI) chatbots in nursing education, focusing on their usage patterns, perceived benefits, and limitations. | 474 nursing faculty members from Jordan and the United States, all with at least one year of teaching experience. | Nursing faculty in Jordan and the U.S(41) |
|  | Majed Mowanes Alruwaili et al. 2024 | Descriptive Cross-Sectional Study | To explore nurses’ awareness and attitudes toward AI and its implications for nursing practice. | 220 Practicing nurses | Saudi Arabia (151) |
