# Supplementary material for: AI literacy and competency in nursing education: preparing students and faculty members for an AI-enabled future-a systematic review and meta-analysis
Source: Front Med (Lausanne). 2025 Nov 26;12:1681784. doi: 10.3389/fmed.2025.1681784 (PMC12689331; doi:10.3389/fmed.2025.1681784)
Supplement: Supplementary file 3 [file Table_3.docx]

Appendix C – Search Strategy for the Systematic Review

| **Database** | **Search String (Boolean Logic)** | **Years Covered** | **Results Retrieved (n)** |
| --- | --- | --- | --- |
| **Google Scholar** | (“Artificial Intelligence” OR “AI” OR “Machine Learning” OR “Deep Learning”) AND (“Nursing Education” OR “Nursing Students” OR “Nursing Faculty”) AND (“Competency” OR “Readiness” OR “AI Literacy”) | January 2020 – June 2025 | 49,056 |
| **Scopus** | (“Artificial Intelligence” AND “Nursing Education”) OR (“AI in Nursing Curriculum”) | January 2020 – June 2025 | 780 |
| **Web of Science** | (“AI” AND “Nursing Education” AND “Competence”) | January 2020 – June 2025 | 690 |
| **PubMed + PubMed Central + Medline** | (“Artificial Intelligence” [Mesh] OR “Machine Learning”) AND (“Nursing Education” [Mesh]) | January 2020 – June 2025 | 3,529 (278 Medline + 3 251 PMC) |
| **Cochrane Library** | (“Artificial Intelligence” AND “Nursing”) | January 2020 – June 2025 | 34 |
| **Semantic Scholar** | (“AI” AND “Nursing Education”) | January 2020 – June 2025 | 332 |
| **CINAHL via EBSCOhost** | (“Artificial Intelligence” OR “Machine Learning”) AND (“Nursing Education” OR “Nurse Educators”) AND (“Curriculum Integration”) | January 2020 – June 2025 | 359 |
